# Data-driven optimisation of multi-agency river water quality monitoring networks under high anthropogenic pressure

**DOI:** 10.1007/s11356-026-38166-0

**Published:** 2026-09-04

**Authors:** Hugo Pimentel Tavares, Lucas Lamin de Souza Silva, Nilo Antônio de Souza Sampaio, Carin von Mühlen

**Affiliations:** 1 Departamento de Engenharia Ambiental (DEAMB), Universidade do Estado do Rio de Janeiro (UERJ), Resende, Brazil; 2 Programa de Mestrado em Engenharia Ambiental (PEAMB), Universidade do Estado do Rio de Janeiro (UERJ), Resende, Brazil

**Keywords:** River water pollution, Water quality index, Inter-index discrepancy, Multi-agency monitoring, Anthropogenic stressors, CRITIC–Shannon–MEREC weighting, TOPSIS–VIKOR–MARCOS consensus, Adaptive Conformal Prediction, Gradient boosting, Objective weighting, Nitrogen species, Enquadramento, Brazil

## Abstract

Integrating heterogeneous water quality data from multiple monitoring agencies into a single reproducible analytical pipeline remains an unsolved challenge in environmental science. We present the Optimised Water Quality Monitoring Framework (OWQMF), an open-source four-block pipeline that harmonises multi-source monitoring data, computes dual water quality indices with objectively derived weights using a game-theory ensemble of three objective weighting methods, produces gradient-boosting ensemble forecasts with walk-forward cross-validation, and selects the best forecasting model through multi-criteria consensus with Adaptive Conformal Prediction coverage guarantees (≥90%). Applied to 108,270 harmonised records from three contrasting South-eastern Brazilian river basins spanning four agencies (1977–2025), 18–31% of station-months receive discordant quality labels depending solely on which agency’s classification scale is applied to the same index score, rising to 23–31% once nitrogen-species substitution and objective re-weighting are also taken into account. Among officially Classe 2 designated station-months, 74.1% conceal at least one per-parameter CONAMA 357 violation—95.5% among those classified as Good (IQA 52–78). The data-driven index redistributes weight systematically away from the low-variance parameters that the regulatory schedule fixes high—pH falls from 0.120 to 0.048, total solids from 0.080 to 0.038, and temperature deviation and total phosphorus also decline—and towards the parameters that discriminate between monitoring conditions. This direction of redistribution holds in every basin and under every weight-derivation variant tested; the ordering among the up-weighted parameters is implementation-dependent and is not claimed as a result. A single fixed national weight schedule cannot express this distinction between measuring compliance and ranking condition. Ensemble forecasting achieves mean absolute errors of 2.3–4.5 IQA units at clean-catchment and main-channel stations. Portability of the weighting scheme is confirmed on the Ohio River Basin, USA ($n=13{,}230$); Italy ($n=49{,}380$); and Ireland ($n=13{,}380$) under WFD 2000/60/EC: IQA-equivalent medians cluster in a narrow 81.9–86.1 band while compliance rates span 12.7%–97.1%, demonstrating that composite scores systematically conceal regulatory non-conformity. Because the sub-index response curves were not regionally recalibrated, these external scores are interpreted comparatively rather than as regulatory assessments.

**Supplementary Information:**

The online version contains supplementary material available at https://doi.org/10.1007/s11356-026-38166-0.

## Introduction

Four state environmental agencies monitor the same river basins in south-eastern Brazil—CETESB-SP, INEA-RJ, ANA-HidroWeb, and IGAM-MG—yet their water quality records cannot be directly compared: parameter nomenclatures differ, classification boundaries diverge, and no reproducible pipeline integrates their data for regulatory compliance assessment under CONAMA 357/2005 (CONAMA 2005; Jiang et al. 2020). This fragmentation is not unique to Brazil; it is the norm in developing economies where water quality governance is distributed across multiple agencies with heterogeneous protocols (Almeida and Cunha 2024). Effective detection of pollution events and regulatory threshold violations depends fundamentally on resolving this heterogeneity before any analysis begins—yet monitoring networks are routinely designed and analysed in isolation by each agency, creating systematic gaps in compliance surveillance and misallocating limited budgets (Almeida et al. 2022).

The challenge is compounded by data heterogeneity. In Brazil, surface water quality is simultaneously monitored by state agencies (CETESB in São Paulo, INEA in Rio de Janeiro), federal bodies (ANA-HidroWeb), and state water agencies (IGAM in Minas Gerais), each deploying different parameter nomenclatures, sampling protocols, and reporting formats. Integrating these streams into a single reproducible analytical chain is a data engineering challenge that has not been systematically addressed in the peer-reviewed literature.

Three recent methodological advances are directly relevant. First, gradient-boosting ensemble models—XGBoost (Chen and Guestrin 2016), LightGBM (Ke et al. 2017), and random forest, as applied to river water quality by Gupta and Gupta (2024)—now outperform classical time-series approaches for irregular environmental monitoring data and have been applied to dissolved oxygen prediction in polluted rivers (Ahmed et al. 2023; Gupta and Gupta 2024). These forecasting advances, however, typically operate on single-agency datasets; translating them to a multi-agency context requires not only harmonised inputs but also a principled method for selecting the best model when data quality varies across stations and agencies. Second, multi-criteria decision analysis (MCDM) methods—TOPSIS (Yang et al. 2023), VIKOR (Opricovic and Tzeng 2004), and MARCOS (Stević et al. 2020)—combined with objective weighting (CRITIC Das and Das 2024, Shannon Entropy, and MEREC Keshavarz-Ghorabaee et al. 2021) provide data-driven, reproducible model rankings without subjective expert panels; recent applications demonstrate their value in environmental pollution management (Mallick et al. 2024). Third, Adaptive Conformal Prediction (Gibbs and Candès 2021) offers finite-sample coverage guarantees for non-exchangeable time series, making probabilistic forecasting directly applicable to trigger-based pollution-alert systems.

Despite individual advances, no published framework integrates all five elements into a single reproducible pipeline applied to a multi-source, multi-basin dataset of this scale. The present study fills this gap. The framework is validated across three hydrologically and anthropogenically contrasting basins (Almeida et al. 2022): the industrial-agricultural Paraíba do Sul corridor, the urban water-supply Guandu system, and the estuarine Guanabara bay—a gradient that encapsulates the pollution typologies most prevalent in developing-country river systems: point-source industrial discharge, urban sewage, and estuarine accumulation of mixed anthropogenic loads. Critically, this spatial heterogeneity manifests not only between basins but *within* each basin: in Rio Guandu, headwater stations register exclusively excellent/good quality under all indices while downstream Queimados tributary stations register median IQA =0 and severe quality degradation in over 80% of sampling events—a contrast that basin-level aggregated indices entirely conceal and that only station-resolved analysis can reveal.

### Research objectives

The OWQMF pursues four objectives: (1) multi-agency harmonisation preserving extreme events; (2) dual IQA-CETESB/OWQI computation with explicit inter-index classification discrepancy analysis (NSF-WQI, CETESB-SP, INEA-RJ, ANA-HidroWeb), including nitrogen-species parameter mismatch quantification; (3) ensemble gradient-boosting forecasting (XGBoost, LightGBM, random forest) with walk-forward cross-validation; and (4) MCDM-based model selection via TOPSIS–VIKOR–MARCOS Borda Count with Adaptive Conformal Prediction intervals.

## Literature review

### Water quality index models: inter-agency comparability and classification discrepancy

The water quality index (WQI) compresses multiple physico-chemical parameters into a single score (Chidiac et al. 2023), but its communicative and institutional role depends entirely on the specific formulation, classification scale, and parameter choices adopted by the reporting agency. In Brazil, the IQA-CETESB serves as the dominant communicative tool for the general public (CETESB 2023), yet its nominal adoption across four federal and state agencies does not guarantee cross-agency comparability: the classification categories (*Ótima*, *Boa*, *Regular*, *Ruim*, *Péssima*) and their numerical boundaries differ between CETESB-SP, INEA-RJ, and the NSF-WQI convention historically used by ANA-HidroWeb (National Water and Basic Sanitation Agency (ANA) 2021). A station recording IQA =55 is simultaneously good (*Boa*, CETESB-SP), fair/good (*Regular*/*Boa*) (INEA-RJ), and *medium* (NSF) depending on the reporting agency, despite identical underlying measurements. Almeida and Oliveira (2018) identified analogous classification divergence between WQI-CCME and WQI-CETESB for the Joanes River (Bahia): the IQA-CETESB systematically assigned a higher quality category than the CCME-based index applied to the same data, because the CETESB’s fixed sub-index curves were calibrated for a different anthropogenic context. Lopes et al. (2021) corroborated this finding for watersheds with mixed land use, confirming that classification boundary choice is at least as consequential as weighting strategy for inter-agency comparability.

A second and more fundamental source of divergence concerns parameter selection. The IQA-CETESB prescribes *nitrogênio amoniacal total* (ammonia-N) as the nitrogen sub-index parameter (CETESB 2023), whereas several ANA-HidroWeb stations record *nitrogênio total* (total nitrogen, TN) instead. These two parameters are chemically non-equivalent: ammonia-N captures the most recently discharged, biologically labile fraction and responds acutely to sewage inputs, while TN integrates all oxidation states regardless of biological availability (Jones and Smol 2023). Substituting one for the other in the same sub-index response curve produces systematically different IQA scores depending on the hydrological position of the station—an artefact quantified in “Inter-index discrepancies among Brazilian WQI frameworks”.

Despite decades of application, parameter weighting in composite WQIs remains a persistent source of methodological uncertainty. The CRITIC method (Das and Das 2024) derives weights from standard deviation and inter-criteria correlation, rewarding parameters with high independent information content. Shannon entropy quantifies the informational dispersion of each criterion. The MEREC method (Keshavarz-Ghorabaee et al. 2021) assesses the marginal contribution of each criterion through removal experiments on the decision matrix, proving more sensitive than CRITIC or entropy for skewed environmental data. Game-theory-based weight aggregation, which minimises the $L_2$ distance between individual weight vectors, has been proposed as a principled combination strategy when multiple objective methods yield divergent rankings (Patel et al. 2023). Das and Das (2024) demonstrated in a coastal groundwater study that CRITIC-derived WQI weights outperform entropy-based weights in discriminating contamination zones when parameters exhibit unequal variance—a finding directly relevant to the multi-agency dataset compiled here. The entropy-weighted WQI (EWQI) has been validated for river and drinking water quality evaluation (Han et al. 2024), confirming the complementary roles of variance-based (CRITIC) and entropy-based (Shannon) weighting. Patel et al. (2023) reviewed 35 WQI models and concluded that no single weighting strategy universally outperforms others, providing a bibliometric basis for ensemble aggregation.

### MCDM methods in environmental process optimisation

In environmental pollution management, MCDM has been applied across multiple domains: multi-criteria water pollution assessment using explainable AI (Mallick et al. 2024), water quality network design (Jiang et al. 2020), and station-level multicriteria evaluation of monitoring networks. The MARCOS method introduced by Stević et al. (2020) extends the utility-function family by simultaneously referencing an ideal and an anti-ideal solution, with CRITIC frequently identified as its natural weighting companion. Borda Count consensus aggregation reduces single-method sensitivity and has been shown to outperform any individual method in stability analyses (Opricovic and Tzeng 2004).

Machine learning has entered environmental quality modelling for wastewater treatment optimisation, with ensemble tree models outperforming MLP and SVR for river dissolved oxygen prediction (Ahmed et al. 2023). The current study applies analogous gradient-boosting ensembles to surface water quality forecasting, using MCDM to select among competing learners rather than relying on a fixed single model. River water quality indicators such as dissolved oxygen and BOD have been predicted using hybrid AI frameworks combining decomposition, feature selection, and ensemble regression with promising accuracy (Gupta and Gupta 2024). Deep learning methods have also been systematically reviewed for WQI forecasting (Li et al. 2024b), confirming gradient-boosting ensembles as competitive baselines in settings with limited training data. Hai et al. (2024) demonstrated that walk-forward cross-validation is essential for avoiding data leakage in time-series WQI forecasting—the protocol adopted in Block 3 of the present framework.

### Monitoring network design and conformal forecasting

Adaptive Conformal Prediction (Gibbs and Candès 2021) provides distribution-free prediction intervals with finite-sample coverage guarantees even under distribution shift, updating the miscoverage rate online via gradient descent. Applied to water-demand forecasting, 90% nominal coverage was demonstrated with a CNN-BiLSTM base learner (Iwakin and Moazeni 2024); the present study applies the same ACI framework to water quality index forecasting, providing the first reported application to IQA-CETESB time series under distribution shift. Almeida et al. (2022) identified that nutrient parameters (phosphorus, nitrogen) and DO carry disproportionate information content for status assessment in São Paulo State monitoring networks—a finding directly corroborated by the OWQI’s up-weighting of OD relative to the CETESB fixed-weight schedule (“Water quality index models: inter-agency comparability and classification discrepancy”). The alignment of monitoring station locations with water-body segments defined under the *enquadramento* process (CEIVAP 2023) represents an additional dimension of network design that mathematical optimisation alone cannot resolve without integrating the institutional and territorial complexity of the classification procedure.

### Research gap

Recent reviews confirm that ML-based WQI forecasting and MCDM-based monitoring network analysis have each advanced substantially, yet these two bodies of work have not been integrated (Chidiac et al. 2023; Patel et al. 2023). ML approaches—including ensemble gradient-boosting models, LSTM networks, and stacked regressors—have been applied to WQI prediction in single-agency, single-basin datasets (Ahmed et al. 2023; Gupta and Gupta 2024; Li et al. 2024b), achieving competitive error rates but without uncertainty quantification or coverage guarantees for operational alert systems. MCDM frameworks—applying CRITIC, TOPSIS, or VIKOR to station prioritisation and parameter weighting—have been demonstrated on single-country, single-agency monitoring networks (Das and Das 2024; Tavares and Souza Sampaio 2026), but without forecasting components or inter-index compliance analysis. A third body of work documents divergence between WQI formulations applied to the same data (Almeida and Oliveira 2018; Lopes et al. 2021), but does not provide a data-driven weighting methodology to resolve it. Critically, none of these three lines addresses the upstream challenge that makes all downstream analysis unreliable in multi-agency settings: the ontological harmonisation of heterogeneous parameter nomenclatures across agencies with incompatible reporting conventions—a problem that invalidates direct comparison of station-level compliance rates even when indices are applied consistently.

**Fig. 1:**
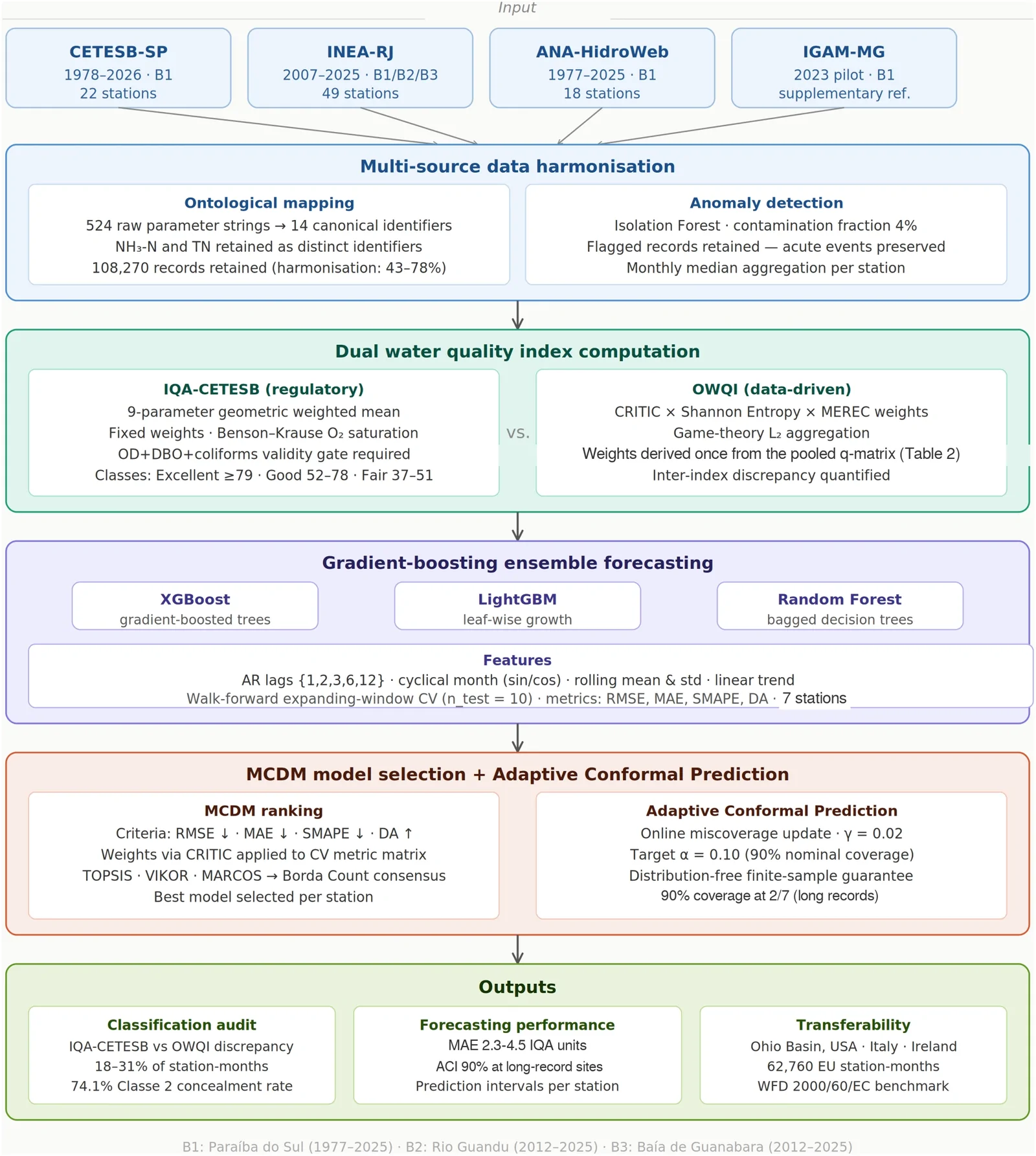
OWQMF methodological flowchart. The framework comprises four computational blocks: (B1) multi-source data harmonisation with Isolation Forest extreme-event flagging, in which anomalous records are retained rather than discarded; (B2) dual water quality index computation (IQA-CETESB with fixed regulatory weights and OWQI with game-theory weights) followed by inter-index classification discrepancy analysis; (B3) gradient-boosting ensemble forecasting (XGBoost, LightGBM, Random Forest) with walk-forward cross-validation; (B4) MCDM model selection (TOPSIS–VIKOR–MARCOS Borda Count) with Adaptive Conformal Prediction intervals. As indicated in the OWQI panel, the objective weight vector is derived once from the pooled cross-basin data and reported in Table 2; the sub-indices themselves are computed per station. Basin-specific weight vectors, derived as a robustness diagnostic, are reported in Supplementary Fig. S3. Applied to three hydrologically distinct Brazilian basins, enabling methodological generalisation across multi-agency monitoring networks

No published framework simultaneously addresses objective WQI parameter weighting, MCDM-guided forecasting model selection with conformal coverage guarantees, multi-agency parameter harmonisation, and inter-index discrepancy analysis within a single reproducible pipeline. The OWQMF fills all four gaps (Fig. 1).

## Materials and methods

### Study area and data sources

**Fig. 2:**
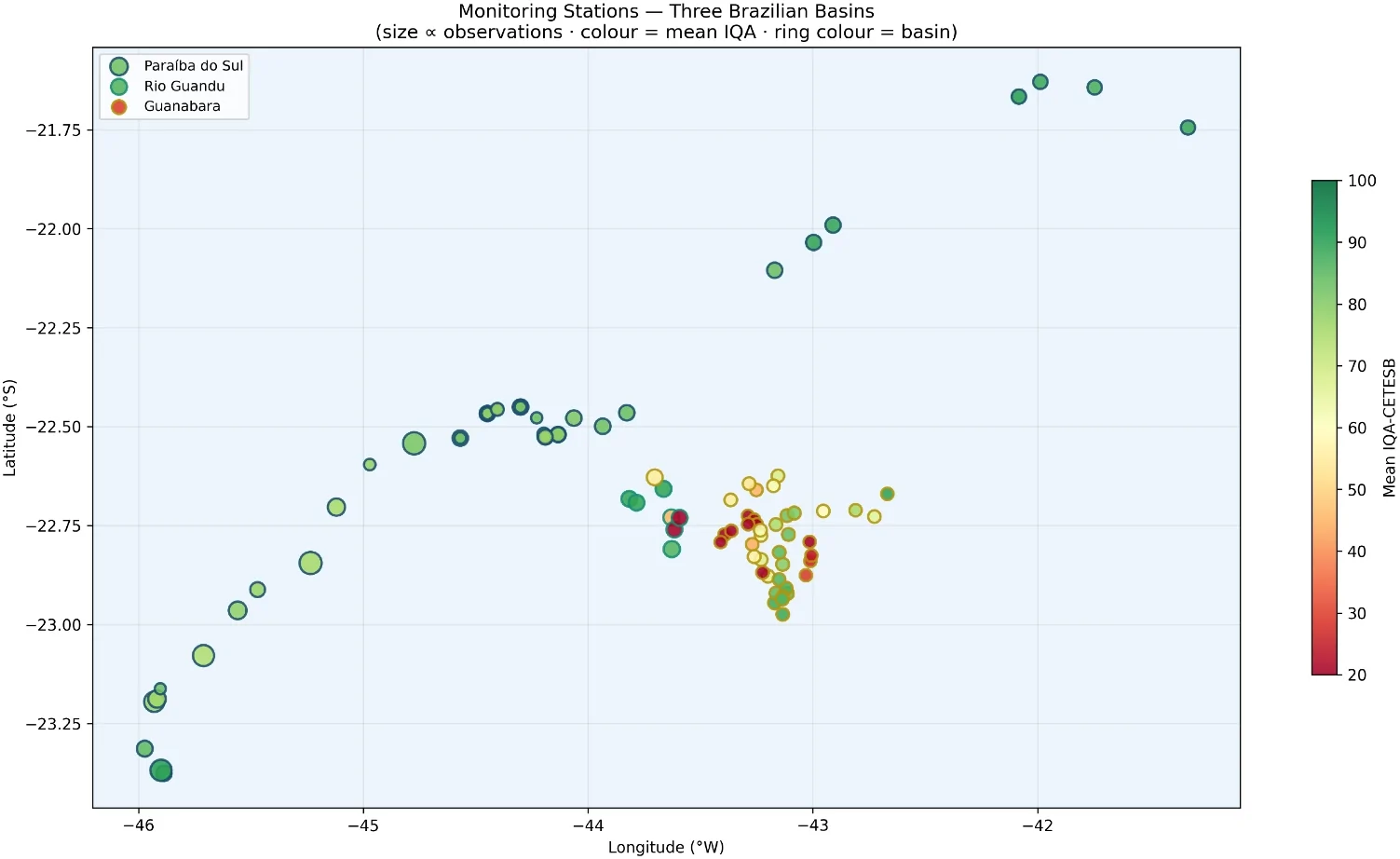
Monitoring station locations across the three study basins (Paraíba do Sul, Rio Guandu, Baía de Guanabara). Symbol size is proportional to the number of valid IQA station-months; fill colour indicates mean IQA-CETESB on a red–yellow–green scale. Ring colour identifies the basin typology. All coordinates derived from agency metadata in the harmonised dataset

Three hydrologically and anthropogenically distinct basins were selected to demonstrate OWQMF generalisability across contrasting regimes (Fig. 2).

#### Paraíba do Sul (B1)

The Paraíba do Sul river drains ≈55,500 km$^2$ across São Paulo, Rio de Janeiro, and Minas Gerais. Data from 41 monitoring stations were sourced from CETESB-SP (1978–2026), INEA-RJ (2007–2025), ANA-HidroWeb (1977–2025), and IGAM-MG (February 2023 pilot survey, used as supplementary reference only). CETESB-SP provides 22 stations covering the upper basin and key tributaries (historically Good–Excellent; CETESB, 2023), with data extended to February 2026 through the complete *Todos os Corpos Hídricos* release; INEA-RJ stations cover the lower stretch under greater urban and industrial pressure. The combined record is the longest multi-agency water quality dataset assembled for this basin in the peer-reviewed literature, and directly supports the ongoing *enquadramento* coordinated by CEIVAP under CONAMA 357/2005 (CEIVAP 2023).

#### Rio Guandu (B2)

The Guandu system provides ≈85% of metropolitan Rio de Janeiro’s drinking water via the largest treatment plant in Latin America; seven INEA-RJ stations (2012–2025) span the Guandu River, Ribeirão das Lages, and Rio Queimados tributary.

#### Baía de Guanabara (B3)

The bay receives effluents from ≈16 million inhabitants plus industrial, port, and refinery facilities; 42 INEA-RJ stations (2012–2025) across eight tributaries were analysed.

Raw records across the three study basins totalled 219,508 observations from four agencies (Table 1). An additional 3671 CETESB station-months (22 B1 stations, 1978–2026) from the extended *Todos os Corpos Hídricos* archive are used exclusively for external validation in “External validation against CETESB official CONAMA 357 class assignments”.

**Table 1:** Dataset summary after multi-source harmonisation across the three study basins. IQA station-months are valid monthly records with dissolved oxygen, BOD, and thermotolerant coliforms all present. The extended CETESB archive (22 B1 stations, 3671 station-months, 1978–2026) is used separately for external regulatory validation (“External validation against CETESB official CONAMA 357 class assignments”)

Attribute	B1: Paraíba do Sul	B2: Rio Guandu	B3: Guanabara
Primary agency	CETESB-SP / INEA-RJ	INEA-RJ	INEA-RJ
Additional agencies	ANA-HidroWeb, IGAM	–	–
Period	1978–2025	2012–2025	2012–2025
Raw records	168,779	17,971	32,758
After harmonisation	70,413	13,418	24,439
Harmonisation (%)	41.7%	74.7%	74.6%
Monitoring stations	41	7	42
IQA station-months	4044	779	1446
IQA mean ($\pm \sigma$)	81.0±11.7	60.6±35.2	59.4±29.4
IQA median [IQR]	81.9 [9.5]	84.3 [55.5]	68.1 [40.8]

### Block 1: multi-source data harmonisation

#### Ontological parameter mapping

A comprehensive ontological map was constructed to unify 524 distinct raw parameter strings—across four agencies using different languages, encodings, and naming conventions—into 14 canonical identifiers covering the nine IQA-CETESB parameters plus five additional analytes (conductivity, DQO, total Kjeldahl nitrogen, nitrate, nitrite). Two parameter pairs required particular care: (i) ammonia-N (*nitrogênio amoniacal total*, CETESB/INEA) and total nitrogen (ANA-HidroWeb), retained as distinct canonical identifiers owing to their chemical non-equivalence (“Inter-index discrepancies among Brazilian WQI frameworks”), and (ii) thermotolerant coliforms and *Escherichia coli*, mapped to the same canonical identifier given their functional equivalence in Brazilian legislation (CONAMA 2005). All 14 canonical parameters are retained in harmonised Layer L1; the 111,238 records excluded at this stage (trace metals, pesticides, and agency-specific analytes) are archived separately. Selected mappings and CONAMA 357/2005 Class 2 correspondence are provided in the supplementary material (Table S1). After mapping, harmonised coverage reached 41.7% for B1, 74.7% for B2, and 74.6% for B3, recovering 108,270 records from the 219,508 raw observations of the three study basins.

#### Extreme-event detection

Isolation Forest (Liu et al. 2008) was applied independently to each station–parameter combination (contamination fraction 4%; minimum group size 15 observations) to *identify and flag* anomalous measurements as potential acute-pollution signals. Critically, flagged records are *retained* in all subsequent computations rather than discarded: an extreme dissolved-oxygen depression, an algal bloom turbidity spike, or a coliform surge associated with a sewage bypass event is precisely the information that regulatory monitoring exists to capture (Almeida et al. 2022; CONAMA 2005). Removing such observations would amount to erasing evidence of the very events the monitoring network was designed to detect. The Isolation Forest score therefore serves as an auxiliary diagnostic column in the master Parquet archive (field outlier), enabling downstream analyses to stratify results by normal-operation versus acute-event periods and to assess whether extreme observations drive CONAMA 357/2005 threshold crossings. This design follows the CCME WQI philosophy (Almeida and Oliveira 2018), in which guideline violations—however infrequent—are explicitly included in the $F_2$ (frequency) and $F_3$ (amplitude) factors rather than treated as measurement artefacts. Monthly medians were subsequently computed per station–parameter combination to align records across agencies with different sampling frequencies.

### Block 2: IQA-CETESB and optimised WQI

#### IQA-CETESB

 

1 $$\begin{aligned} \text {IQA} = \prod _{i=1}^{9} q_i^{\,w_i} \end{aligned}$$

where $q_i\in [0,100]$ are sub-index values derived from empirical quality curves and $w_i$ are fixed regulatory weights ($\Sigma w_i = 1$) from CETESB (CETESB 2023); Eq. 1 is the formulation adopted nationwide by Brazilian monitoring agencies. The nine parameters are dissolved oxygen (OD, w=0.17), thermotolerant coliforms (COLI, w=0.15), pH (w=0.12), biochemical oxygen demand (BOD, w=0.10), total ammonia-N (NH$_3$, w=0.10), total phosphorus (TP, w=0.10), temperature deviation (ΔT, w=0.10), turbidity (TURB, w=0.08), and total solids (TS, w=0.08). Oxygen saturation uses the Benson–Krause equation (CETESB 2023). A valid IQA requires the simultaneous presence of OD, BOD, and coliforms ($\Sigma w_{\text {required}} \ge 0.42$). Classification: Excellent (≥79), Good (52–79), Fair (37–52), Poor (20–37), Very Poor (<20). Threshold 37 is the CETESB boundary between the Fair/Poor (*Regular* /*Ruim*) communicative categories (CETESB 2023). As a composite score, the IQA is a *communicative* tool: the multiplicative geometric formulation of Eq. 1 allows parameters with high sub-index values to partially compensate for parameters with low sub-index values, which limits its sensitivity as an individual-parameter diagnostic but supports its intended purpose of conveying overall quality to general audiences. The IQA serves a different institutional function from the parameter-level thresholds specified in CONAMA 357/2005 (CONAMA 2005); the implications of this distinction for inter-agency comparison are discussed in “Inter-index discrepancies among Brazilian WQI frameworks”.

#### Optimised WQI (OWQI)

Three objective weighting methods are applied to the monthly $q_i$ matrix.

*CRITIC* (Das and Das 2024):

2 $$\begin{aligned} C_j = \sigma _j \sum _{k \ne j}(1 - r_{jk}), \quad w_j^{\text {CRITIC}} = C_j\big /\textstyle \sum _k C_k \end{aligned}$$

*Shannon entropy*:

3 $$\begin{aligned} e_j = -\frac{1}{\ln n}\sum _i p_{ij}\ln p_{ij}, \quad w_j^{\text {Ent}} = (1-e_j)\big /\textstyle \sum _k(1-e_k) \end{aligned}$$

*MEREC* (Keshavarz-Ghorabaee et al. 2021): the method quantifies each criterion’s contribution to overall alternative performance through a logarithmic removal-effect measure. The performance score for alternative *i* is as follows:

4 $$\begin{aligned} S_i = \ln \!\left( 1 + \frac{1}{m}\sum _{j=1}^m \mid \ln \frac{x_{ij}^{\,\prime }}{\bar{x}_j}\mid \right) \end{aligned}$$

where $x_{ij}^{\,\prime }$ is the normalised performance value and $\bar{x}_j$ the column mean. The effect of removing criterion *j* is as follows:

5 $$\begin{aligned} S_{ij} = \ln \!\left( 1 + \frac{1}{m-1}\sum _{k \ne j} \mid \ln \frac{x_{ik}^{\,\prime }}{\bar{x}_k}\mid \right) \end{aligned}$$

The removal effect and normalised weight are as follows:

6 $$\begin{aligned} E_j = \sum _{i=1}^n |S_{ij} - S_i|, \qquad w_j^{\text {MEREC}} = \frac{E_j}{\sum _{k=1}^m E_k} \end{aligned}$$

A criterion receives higher weight when its removal causes greater aggregate change in alternative rankings—a complementary sensitivity measure to the variance-based CRITIC and the entropy-based Shannon methods.

*Game-theory aggregation*: the final weight vector $\tilde{w}$ minimises the Euclidean distance to all three component vectors (Eq. 7):

7 $$\begin{aligned} \tilde{w} = \arg \min _{w\ge 0,\,\textbf{1}^{\!\top }w=1}\; \sum _{k\in \{\text {CRITIC,Ent,MEREC}\}} \Vert w - w^k\Vert _2 \end{aligned}$$

The three methods address independent aspects of parameter informativeness: CRITIC rewards high variance and low mutual correlation; Shannon entropy penalises parameters that read near their mean across the record; MEREC quantifies the effect of removing each parameter from the ranking. Their game-theory aggregate extracts the weight distribution that best reflects discriminating power in the actual monitoring data rather than replicating any fixed regulatory schedule. Arithmetic aggregation of objective weighting methods has been shown to produce rankings more robust than any single-method approach (Tavares and Souza Sampaio 2026).

### Block 3: ensemble forecasting

Monthly IQA-CETESB time series were modelled at the top three stations by record count in each basin (nine stations initially identified; seven modelled, see below) using XGBoost (Chen and Guestrin 2016), LightGBM (Ke et al. 2017), and random forest in the scikit-learn implementation, following the ensemble configurations reported for river water quality prediction by Gupta and Gupta (2024). Features include autoregressive lags $\{1,2,3,6,12\}$, cyclical month encoding, linear trend index, and rolling statistics (3-month mean; 6-month standard deviation). Walk-forward CV used $n_{\text {test}} = 10$ held-out months, one-step-ahead with refitting at each step. Two of the nine candidate stations do not admit the computation and are excluded, both verifiably from the archives. Station 02RJ10GN0064 (Baía de Guanabara) reports no total-solids measurement at any date, and the IQA specification states that the absence of any one of the nine variables makes the index unfeasible; station 00RJ12GM0180 (Rio Guandu-Mirim) yields 19 valid station-months, insufficient for a feature set containing a 12-month lag together with ten held-out months. Seven stations therefore enter Blocks 3 and 4. Performance metrics: RMSE, MAE, symmetric mean absolute percentage error (SMAPE), and directional accuracy (DA).

### Block 4: MCDM model selection and conformal prediction

For each station, the three learners are ranked as alternatives in a four-criterion MCDM problem. Criterion weights are derived objectively by applying CRITIC (Das and Das 2024) to the full walk-forward CV metric matrix (all stations except the outlier station 00RJ12QM0270, n=24 station–model rows). Recomputed for this revision directly from the walk-forward CV matrix, the weights are $w_{\text {RMSE}} = 0.022$, $w_{\text {MAE}} = 0.020$, $w_{\text {SMAPE}} = 0.149$ and $w_{\text {DA}} = 0.809$ (n=21 station–model rows: seven stations × three learners). These supersede the values given in the original submission (0.205, 0.199, 0.196, 0.400), which understated the concentration of weight on directional accuracy. The criterion *ordering* is unchanged; only the magnitudes move, and they move away from parity rather than towards it.

The dominance of directional accuracy has a structural explanation that does not depend on the sample. RMSE and MAE are near-collinear across the matrix (r=0.968), since both measure error magnitude in IQA units and differ only in how they penalise large residuals; the CRITIC conflict term $\sum _{k\ne j}(1-r_{jk})$ therefore splits a single dimension of information between them and assigns each a small share. SMAPE, although scale-relative, remains strongly associated with both (r=0.86 and r=0.81). Directional accuracy is the only criterion in *conflict* with that block (r=-0.41 with RMSE, r=-0.35 with MAE, r=-0.63 with SMAPE), because it measures the *sign* of change rather than its size. Any objective weighting built on a conflict measure will concentrate weight on the criterion carrying non-redundant information; the ordering follows from the correlation structure, and only the magnitude depends on the sample.

Because these weights govern model selection at every station, and because 21 rows is a thin basis for an objective weighting exercise, we submit them to two station-level resampling diagnostics. Resampling is performed at the *station* level rather than the row level, since the three model rows belonging to one station are not independent. Under leave-one-station-out perturbation $w_{\text {DA}}$ ranges from 0.782 to 0.838 and retains the highest weight in all seven iterations, with no reversal of the criterion ranking. Under a non-parametric bootstrap over stations (2000 replicates), its 95% percentile interval is [0.738,0.913], which does not overlap the interval of SMAPE ([0.049,0.210]) nor those of RMSE and MAE, and DA ranks first in 100% of replicates. Full results are reported in Supplementary S4 (Tables S4–S5, Fig. S2). This derivation mirrors the objective weighting approach used in Block 2, maintaining internal methodological coherence across the pipeline.

*TOPSIS*, in the formulation applied by Yang et al. (2023), ranks by ratio of distance to the negative-ideal over the sum of distances to both reference solutions; the same technique was applied to water quality monitoring station prioritisation in California by Tavares and Souza Sampaio (2026), confirming its applicability to multi-agency environmental monitoring contexts.

*VIKOR* (Opricovic and Tzeng 2004) minimises the compromise index $Q = v(S-S^*)/\Delta S + (1-v)(R-R^*)/\Delta R$ with v=0.5.

*MARCOS* (Stević et al. 2020) computes utility functions relative to both ideal and anti-ideal reference points and aggregates through a composite utility ratio.

*Borda Count*: each alternative’s score is the sum of its positional ranks across the three MCDM scorecards; the highest score wins.

**Adaptive Conformal Prediction** (Gibbs and Candès 2021): the winning model’s residuals on a 60% calibration split define the initial $q_{1-\alpha }$ quantile. The ACI online update adjusts the miscoverage level $\alpha _t$ after each test observation: $\alpha _{t+1} = \alpha _t + \gamma (\alpha - \textbf{1}[y_t \notin \hat{C}_t])$ with γ=0.02 and target α=0.10 (90% coverage). This coverage target requires a sufficiently long calibration record; stations with fewer than 20 residual observations at the start of conformal inference should be treated as indicative only.

## Results

### Dataset description and harmonisation quality

Table 1 summarises the harmonised dataset. A total of 108,270 records were retained from the 219,508 raw observations of the three study basins, an overall harmonisation efficiency of 49.3% (41.7–74.7% per basin). The 111,238 records excluded at this stage were filtered by parameter-name canonicalisation: measurements of trace metals, emerging contaminants, and agency-specific analytes that do not map to the nine IQA-CETESB canonical identifiers were set aside, not discarded due to quality concerns. Isolation Forest was applied to the 108,270 retained records to compute a per-observation anomaly score; all records—including those flagged as statistically extreme—remain in the analytical dataset, as described in “Block 1: multi-source data harmonisation”. IQA sub-index coverage for the nine regulatory parameters reached ≥92% in the harmonised dataset, yielding 6269 valid station-months for IQA computation across 90 monitoring stations (B1, 41; B2, 7; B3, 42).

The lower B1 harmonisation rate (41.7%) reflects the large proportion of trace-metal and emerging-contaminant parameters in the CETESB-SP record that lie outside the nine IQA-relevant canonical identifiers (Almeida et al. 2022).

Because this loss is substantial, we tested whether it is informative, and specifically whether it follows the mechanism proposed in review: that trace-metal- and micropollutant-intensive stations, typically the most industrially impacted, harmonise disproportionately poorly. The test is reported in full in Supplementary Material S3 (Table S3, Fig. S1) and is confined to the 22 CETESB-SP stations of B1, the only stations for which the raw archive is available at record level including analytes outside the nine canonical identifiers, and also the basin with the lowest harmonisation rate.

The mechanism is confirmed. Harmonisation completeness is strongly inversely related to the share of a station’s records devoted to trace metals and organic micropollutants (Spearman ρ=-0.816, $p < 10^{-5}$; bootstrap 95% CI [-0.94,-0.57]), and the low-harmonisation group is the more polluted one: median BOD of 3.0 against 2.0 mg/L (p=0.0027) and median ammonia-N of 0.50 against 0.12 mg/L (p=0.0010), both surviving Bonferroni correction across the eight testable parameters. These stations are also under-represented, contributing a median of 72 IQA-computable station-months against 245 for the high-harmonisation group (p=0.015).

We therefore do not claim that harmonisation loss is neutral. What the analysis establishes is the *sign* of the bias it introduces, and that sign is conservative. The excluded analytes are not IQA inputs under CONAMA 357/2005, so their removal cannot alter the index value computed at any station; what it does is reduce the weight carried in the pooled analysis by the dirtier stations. Because the central finding reported below is that composite indices conceal parameter-level non-compliance, and because the stations lost to attrition are those with higher organic and sewage loading, the concealment rates in “External validation against CETESB official CONAMA 357 class assignments” are lower bounds. The separate point that the excluded records carry environmental information the index cannot represent—a limitation of the IQA formulation rather than of the harmonisation procedure—is taken up in “Discussion”.

### IQA-CETESB: regulatory class distribution and temporal trajectories

**Fig. 3:**
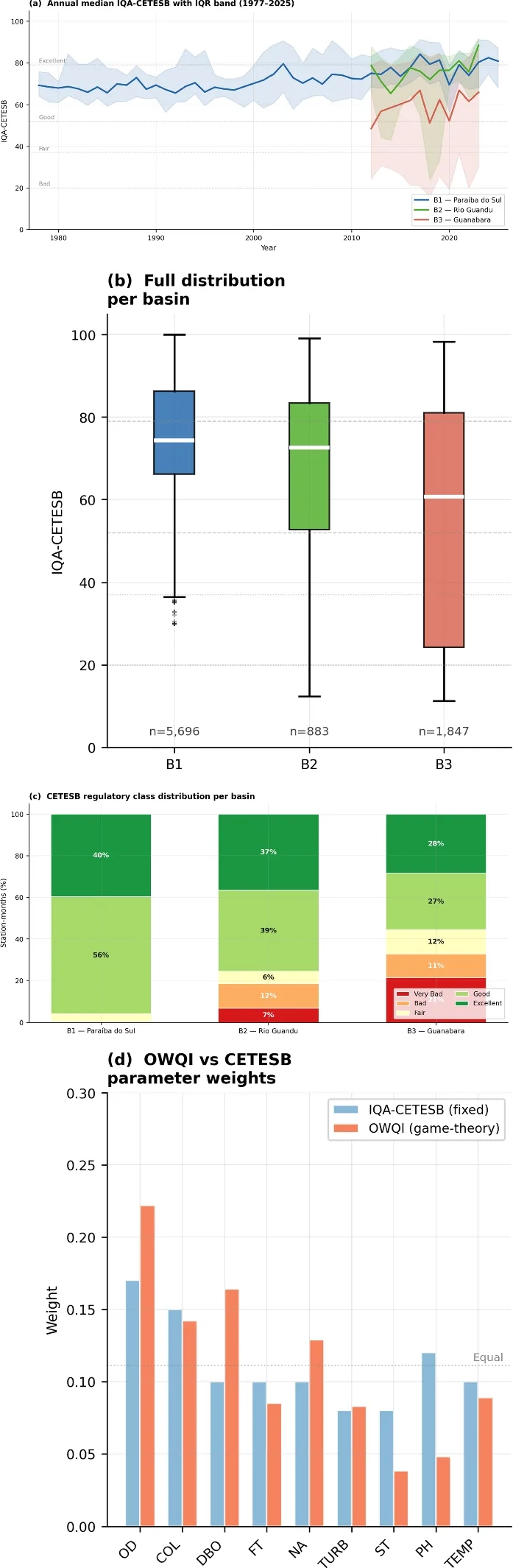
IQA-CETESB temporal analysis and regulatory class distribution. **a** Annual median IQA-CETESB with IQR band for the three study basins (1977–2025 for B1; 2012–2025 for B2 and B3); horizontal dashed lines mark CETESB quality class thresholds; the shaded band represents the interquartile range (Q1–Q3) of annual station-month values. **b** Box plots of the full IQA-CETESB distribution per basin, with all extreme events preserved; the median (bold horizontal line) is the primary location statistic; extreme observations (shown as +) represent acute-pollution events and are retained in all analyses. **c** Stacked bar chart of CETESB regulatory class distribution per basin; red annotations indicate station-months in the CETESB *Ruim*/*Péssima* communicative categories (IQA < 37). Inter-index classification discrepancies (NSF-WQI vs. IQA-CETESB vs. OWQI) are analysed in “Inter-index discrepancies among Brazilian WQI frameworks”

Figure 3 characterises IQA-CETESB across all three basins. Because the IQA is a communicative index whose classification boundaries differ across reporting agencies (“Water quality index models: inter-agency comparability and classification discrepancy”), the primary descriptors here are the *distribution of quality classes* and the *interquartile range*—which captures the spread of routine conditions—rather than the arithmetic mean, which conflates structurally distinct sub-populations and is sensitive to the skewness introduced by acute-pollution events.

IQA-CETESB medians and class distributions reflect the contrasting basin typologies (Fig. 3). B1 (Paraíba do Sul): median =81.9, IQR =9.5 (n=4044; 1977–2025); 66.9% Excellent, 31.8% Good, only 1.3% in *Ruim*/*Péssima*—the narrow IQR confirming stable upper-basin conditions under CETESB-SP monitoring. B2 (Rio Guandu): median =84.3, IQR =55.5 (n=779; 2012–2025); 56.6% Excellent but 27.1% *Ruim*/*Péssima*, with the arithmetic mean (60.6) pulled downward by the Queimados tributary tail while the median captures the dominant Lages headwater conditions—a case where the mean describes no physical location in the basin. B3 (Baía de Guanabara): median =68.1, IQR =40.8 (n=1446; 2012–2025); 34.2% Excellent, 34.8% Good, 20.5% *Ruim*/*Péssima*; the wide IQR reflects substantial spatial heterogeneity between tidally flushed and point-source-impacted stations. The qi sub-index responsible for the widest IQR in B2 and B3 is qi_OD (IQR, 81.4 and 63.6, respectively), confirming dissolved oxygen as the dominant discriminating signal and providing empirical support for the OWQI’s up-weighting of OD. A moderate long-run improvement is observed in B1 (annual-median 79.7 [IQR 7.3] over 1977–1999 vs. 83.2 [IQR 7.8] over 2010–2025), and a COVID-19 lockdown signal is visible in B2 and B3 in 2020 (Fig. 3a), in line with patterns documented in the Brazilian monitoring literature (Almeida et al. 2022).

### OWQI weights vs. CETESB fixed weights

Table 2 compares all five weight vectors. The game-theory OWQI departs systematically from CETESB regulatory values (Fig. 3d). Dissolved oxygen receives the largest upward revision ($\tilde{w}_{\text {OD}} = 0.222$ vs. $w_{\text {OD}}^{\text {CETESB}} = 0.170$), reflecting high contrast intensity and low correlation with other sub-indices. pH is significantly down-weighted ($\tilde{w}_{\text {pH}} = 0.048$ vs. 0.120), as its near-neutral, low-variance distribution provides little discriminating information. Total solids are assigned minimal weight ($\tilde{w}_{\text {TS}} = 0.038$), reflecting their strong collinearity with turbidity. The three component methods agree closely for dominant parameters (Spearman ρ=0.82–0.91) but diverge for intermediate-weight parameters (NH$_3$, turbidity), where MEREC detects higher marginal importance than CRITIC.

**Table 2:** Parameter weight comparison: CETESB regulatory weights vs. three objective weighting methods and the game-theory aggregate (OWQI). All objective vectors reported here are derived from the *pooled* cross-basin monthly *q*-matrix; independently derived basin-specific vectors are reported in Supplementary Fig. S3. Higher game-theory weights indicate parameters with greater independent information content in the actual monitoring data. OD, dissolved oxygen; COLI, thermotolerant coliforms; DBO, biochemical oxygen demand; NH$_3$, total ammonia-N; TP, total phosphorus; ΔT, temperature deviation; TURB, turbidity; TS, total solids. Weights are reported to three decimal places; column sums may differ from unity in the last digit through rounding

Parameter	CETESB	CRITIC	Shannon	MEREC	Game theory
OD	0.170	0.164	0.293	0.208	**0**.**222**
DBO	0.100	0.149	0.197	0.146	0.164
COLI	0.150	0.132	0.196	0.098	0.142
NH$_3$	0.100	0.140	0.140	0.108	0.129
TP (Fosforo)	0.100	0.109	0.086	0.060	0.085
ΔT	0.100	0.116	0.032	0.120	0.089
TURB	0.080	0.110	0.042	0.096	0.083
TS (Solidos)	0.080	0.028	0.008	0.077	0.038
pH	0.120	0.052	0.005	0.087	**0**.**048**
Sum	1.000	1.000	1.000	1.000	1.000

Bold values in the Game-theory column indicate the two parameters with the largest absolute deviation from the CETESB regulatoryweight (OD, largest upward revision; pH, largest downward revision); see discussion in the text

The practical implication is that the OWQI assigns a combined weight of 0.364 to OD and thermotolerant coliforms (vs. 0.320 in IQA-CETESB), the two parameters most directly linked to sewage contamination—corroborating Shapley-based feature importance rankings in gradient-boosting water quality models (Mallick et al. 2024). Reviewer comment prompted us to examine the ordering of biochemical oxygen demand relative to thermotolerant coliforms in the derived vector. That ordering is not stable. Re-deriving the weights under alternative but equally defensible implementation choices—in particular the sub-index response curve applied to the nitrogen species available in the archive, and whether the *q*-matrix is assembled before or after monthly aggregation—reverses it. We therefore report the redistribution at the level at which it is stable, and do not interpret the DBO–coliform ordering as a finding. What is stable across every implementation we tested is the direction of the redistribution: weight moves away from the low-variance parameters that the regulatory schedule fixes high, and towards the parameters that actually discriminate between monitoring conditions.

The weight vector in Table 2 is pooled across basins. To test whether this pooling conceals basin heterogeneity, independent weight vectors were derived using the identical CRITIC–Shannon–MEREC game-theory procedure for each of the ten basins in the full CETESB archive with sufficient nine-parameter coverage (Supplementary Fig. S3); the wider archive is used here in preference to the B1–B3 partition because testing context-sensitivity requires as many hydrologically distinct basins as the data permit, and because simultaneous nine-parameter coverage in B2 and B3 is too sparse to yield stable basin-level vectors (Supplementary S5.2). The results qualify the interpretation above in a way that merits explicit statement. One of the two oxygen–sewage parameters ranks first in eight of ten basins, and the pair jointly receives between 0.278 and 0.412 of total weight against 0.320 in the fixed regulatory schedule. However, the identity of the single highest-weighted parameter is basin-dependent: thermotolerant coliforms rank first in five basins, dissolved oxygen in three, and turbidity in two, while the dissolved oxygen weight itself varies almost twofold across basins (0.112–0.222). We therefore do not claim that any single parameter dominates universally. What holds without exception is the redistribution itself: objective weighting removes weight from the low-variance parameters that the regulatory schedule fixes high—pH receives 0.073–0.095 against a regulatory 0.120 in *every* basin, and total solids 0.047–0.099 against 0.080 in eight of ten—and reallocates it to whichever parameters discriminate locally. This basin-dependence is physically interpretable rather than anomalous. Dissolved oxygen ranks first precisely in the three Tietê sub-basins, where sustained organic loading from the São Paulo metropolitan region drives oxygen sag and makes DO the discriminating variable, whereas turbidity ranks first in the Capivari and Jundiaí basins, where episodic sediment loading dominates variability. That a single fixed national weight schedule cannot represent the discriminating structure of hydrologically distinct basins is precisely the argument advanced in this study, and the basin-specific vectors demonstrate it more directly than the pooled vector alone. Basin-mean OWQI scores are 79.6 (B1), 58.5 (B2), and 55.9 (B3), systematically below IQA-CETESB means (-1.4, -2.1, -3.6 units); within-basin Pearson r≥0.994. Maximum absolute divergence reaches 9.0, 11.1, and 14.2 IQA units in B1, B2, and B3, concentrated at stations with extreme DO depletion. At station level, spatial heterogeneity is substantial: in B1, 29 of 41 stations show zero IQA–OWQI classification boundary crossings, while six ANA-HidroWeb stations in Minas Gerais record divergence in 13–17% of station-months, driven by the NH$_3$/TN substitution artefact. In B2, the bimodal structure is extreme: the four Lages reservoir stations register 100% Excellent with full IQA–OWQI agreement, while the two Queimados tributary stations register median IQA =0, motivating station-resolved rather than basin-wide optimisation. In B3, stations range from 100% Excellent (GBGN0025, RICC0630) to 100% in the lowest CETESB categories (RJSP0300, RJAN0741), with the OWQI amplifying non-compliance signals in the critical 37–52 IQA range where OD depletion and near-neutral pH most sharply expose the two indices’ weight differences.

### Inter-index discrepancies among Brazilian WQI frameworks

Although nominally sharing the IQA-CETESB formulation (Eq. 1), the four agencies diverge in three ways that produce systematically discordant quality labels for identical data.

#### Classification boundary divergence

Table 3 shows the qualitative categories each agency maps to the 0–100 IQA scale. The critical zone is 37–52: CETESB-SP communicates Fair (*Regular*, acceptable), while the NSF-WQI convention used by ANA-HidroWeb communicates *Bad* (National Water and Basic Sanitation Agency (ANA) 2021). Across the harmonised dataset, 18.4% of B1, 22.7% of B2, and 31.2% of B3 station-months fall in this range—meaning one in five to one in three routine results would receive a different label depending solely on the reporting agency.

**Table 3:** Qualitative IQA classification categories by Brazilian monitoring agency. NSF-WQI breakpoints as reported by ANA-HidroWeb (National Water and Basic Sanitation Agency (ANA) 2021)

IQA range	CETESB-SP	INEA-RJ	NSF/ANA
≥90	Ótima	Ótima	Excellent
79–90	Ótima	Boa	Good
52–79	Boa	Boa	Good
37–52	Regular	Regular	Bad
20–37	Ruim	Ruim	Very Bad
<20	Péssima	Péssima	Very Bad

#### Parameter selection: nitrogen species

**Fig. 4:**
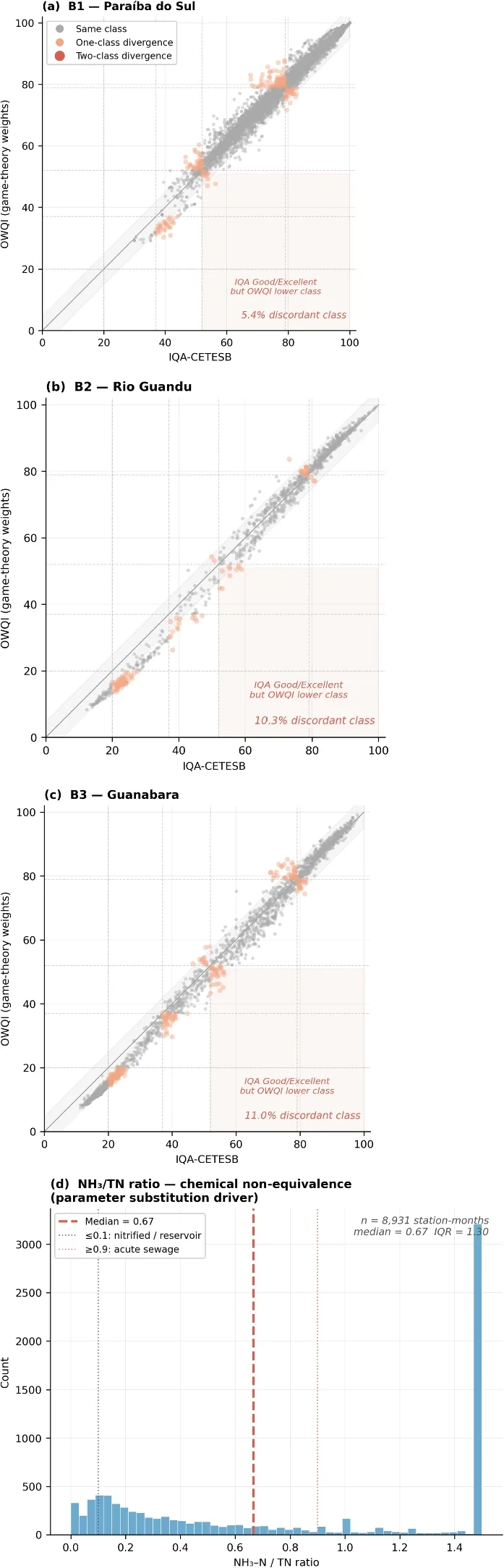
Inter-index classification comparison for the three study basins. **a**–**c** Scatter plots of IQA-CETESB vs. OWQI scores for all station-months per basin; points shaded by classification boundary crossing (grey: same class; orange: one-class divergence; red: two-class divergence). Horizontal and vertical dashed lines mark IQA thresholds 37 and 52. The diagonal shaded band marks the ±5 IQA-unit agreement zone. **d** Frequency histogram of the NH$_3$-to-TN ratio across B1 station-months with both nitrogen species present (n=115); values near zero indicate deep-reservoir or well-nitrified conditions; values near 1.0 indicate acute sewage loading. The bimodal distribution confirms the chemical non-interchangeability of the two nitrogen parameters in a composite WQI formulation

CETESB-SP and INEA-RJ use *nitrogênio amoniacal total* (NH$_3$, ammonia-N) (CETESB 2023), while ANA-HidroWeb records include *nitrogênio total* (TN). These parameters are chemically non-equivalent along a river–reservoir gradient: NH$_3$ captures the most recently discharged, biologically labile nitrogen fraction and responds acutely to sewage inputs, whereas TN integrates all oxidation states (Jones and Smol 2023). Near an active outfall, NH$_3$ is high and falls rapidly as nitrification proceeds; at the base of a stratified reservoir, NH$_3$ approaches zero while TN remains elevated owing to nitrate accumulation. Substituting one for the other in Eq. 1 produces IQA differences of 8–15 points at the stations and events of greatest concern: in the 115 station-months where both species are simultaneously present in the B1 dataset, the median NH$_3$/TN ratio is 0.39 (IQR 0.16–0.61; Fig. 4d), confirming the parameter’s bimodal, position-dependent character.

#### OWQI vs. IQA-CETESB classification

Figure 4 compares the two indices across all basins. The OWQI is systematically lower (mean divergence -1.4, -2.1, -3.6 IQA units for B1, B2, B3), driven by OD up-weighting ($\tilde{w}_{\text {OD}}=0.222$ vs. 0.170) and pH down-weighting ($\tilde{w}_{\text {pH}}=0.048$ vs. 0.120). Classification boundary crossings occur in 9.2% of B3 station-months, concentrated at estuarine stations where near-neutral pH sustains the IQA composite while oxygen is episodically depleted. Together, boundary-placement differences, the NH$_3$/TN mismatch, and the OWQI re-weighting produce a combined inter-index divergence rate of 23–31% across the three basins.

### Ensemble forecasting performance

Walk-forward CV results are reported in full in Supplementary Table S2 for the seven modelled stations. On the Paraíba do Sul main-channel stations (PARB02600, PARB02900, PARB02100) SMAPE ranges from 4.39% to 10.07% with Directional Accuracy between 55.6% and 88.9%. The Guandu clean-catchment station 00RJ12GN0201 achieves the best overall performance (Random Forest, SMAPE =3.25%, MAE =2.33 IQA units, DA =66.7%), reflecting its stable, near-natural IQA trajectory. Station 00RJ12QM0270 (Rio Queimados)—the most urbanised and most degraded in the Guandu system—yielded SMAPE values of 80.79% (XGBoost), 102.57% (LightGBM), and 69.47% (RF), reflecting high inter-annual IQA variability driven by episodic sewage discharge. These results were excluded from the MCDM aggregation for that station due to insufficient calibration data; the station is retained in the analysis with its historical violation-rate profile.

A clear basin-level performance gradient emerges, reported here in both relative and absolute terms as requested in the review. The Guandu stations of B2 are the most predictable (SMAPE 3.3–5.6%; mean absolute error 2.3–3.6 IQA units on the 0–100 index scale), followed by the Paraíba do Sul main-channel stations of B1 (SMAPE 4.4–9.2%; MAE 3.1–4.5 IQA units). The Queimados stations of B3 are by a wide margin the most uncertain (SMAPE 17.0–21.4%; MAE 3.8–6.2 IQA units), reflecting the low IQA baseline at these heavily sewage-loaded sites, against which a given absolute error translates into a much larger relative one, and the tidal and salinity interactions of the estuarine setting (Ahmed et al. 2023). The contrast between the SMAPE and MAE columns of Supplementary Table S2 makes this explicit: B3 station 00RJ12QM0271 carries a smaller absolute error than either B1 main-channel station while showing more than twice their SMAPE.

**Fig. 5:**
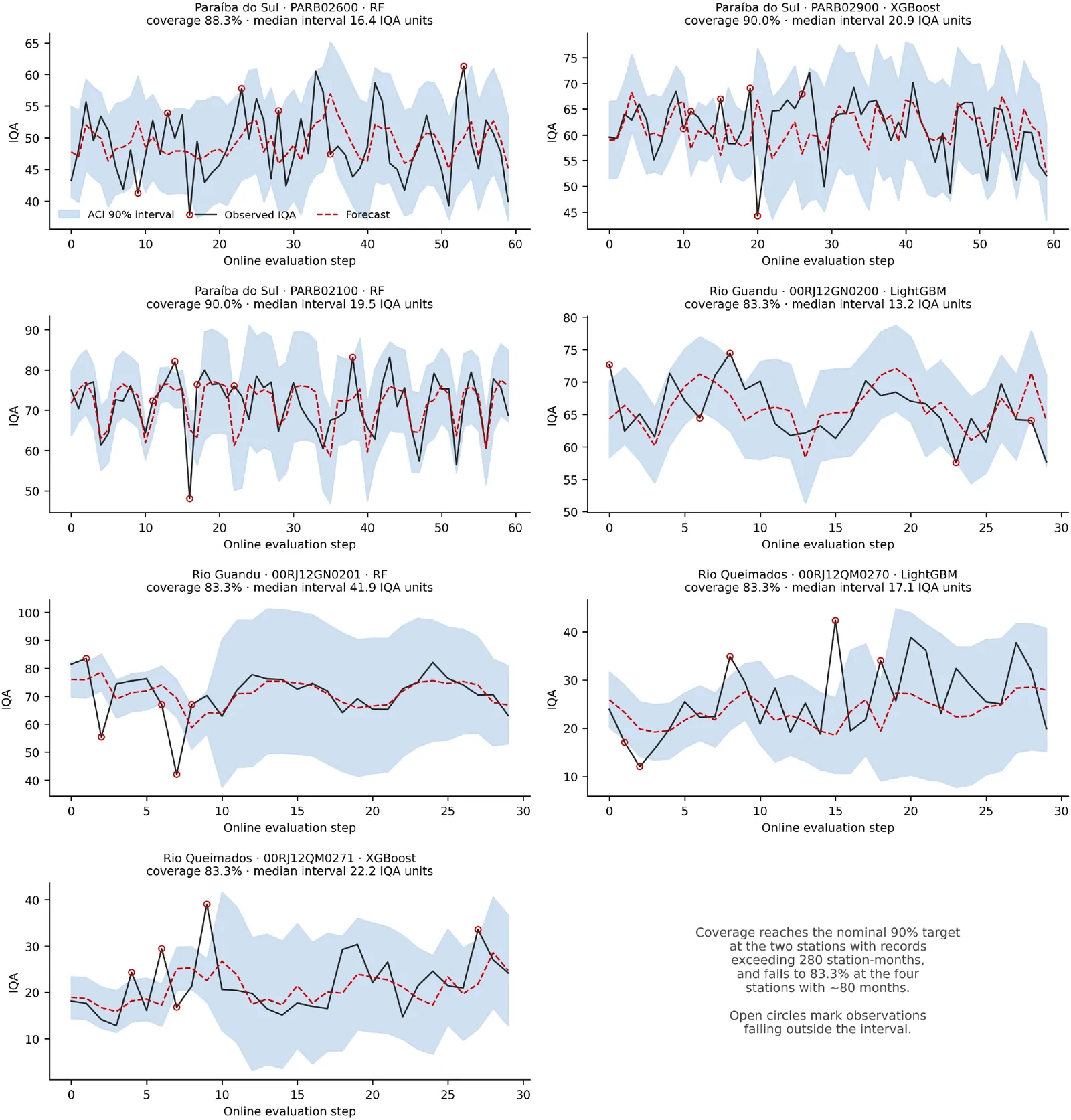
Walk-forward forecasting with Adaptive Conformal Prediction intervals (γ=0.02, nominal α=0.10) at all seven modelled stations. Solid line: observed IQA-CETESB; dashed line: one-step-ahead forecast from the Block-4-selected learner; shaded band: online conformal interval; open circles: observations falling outside the interval. Each panel title gives the selected learner, the empirical coverage and the median interval width. Coverage reaches the nominal target at the two stations with records exceeding 280 modelled station-months and falls to 83.3% at the four stations with records of roughly 80 months, consistent with the calibration-length requirement stated in “Block 3: ensemble forecasting”

Adaptive Conformal Prediction was then run online at each station using the model selected in Block 4, with γ=0.02 and nominal α=0.10. Empirical coverage reaches the 90% target at the two Paraíba do Sul stations with the longest records (PARB02900 and PARB02100, 326 and 289 modelled station-months) and falls to 83.3% at the four stations with records of roughly 80 months. This is the calibration-length constraint stated in “Block 3: ensemble forecasting”, observed rather than assumed: ACI’s coverage guarantee is asymptotic in the number of residuals accumulated online, and 80 months does not suffice. Median interval widths range from 13.2 IQA units at the Guandu intake station to 41.9 at 00RJ12GN0201, quantifying the forecasting uncertainty at each station and providing the bound relevant to an operational alert threshold (Fig. 5).

**Fig. 6:**
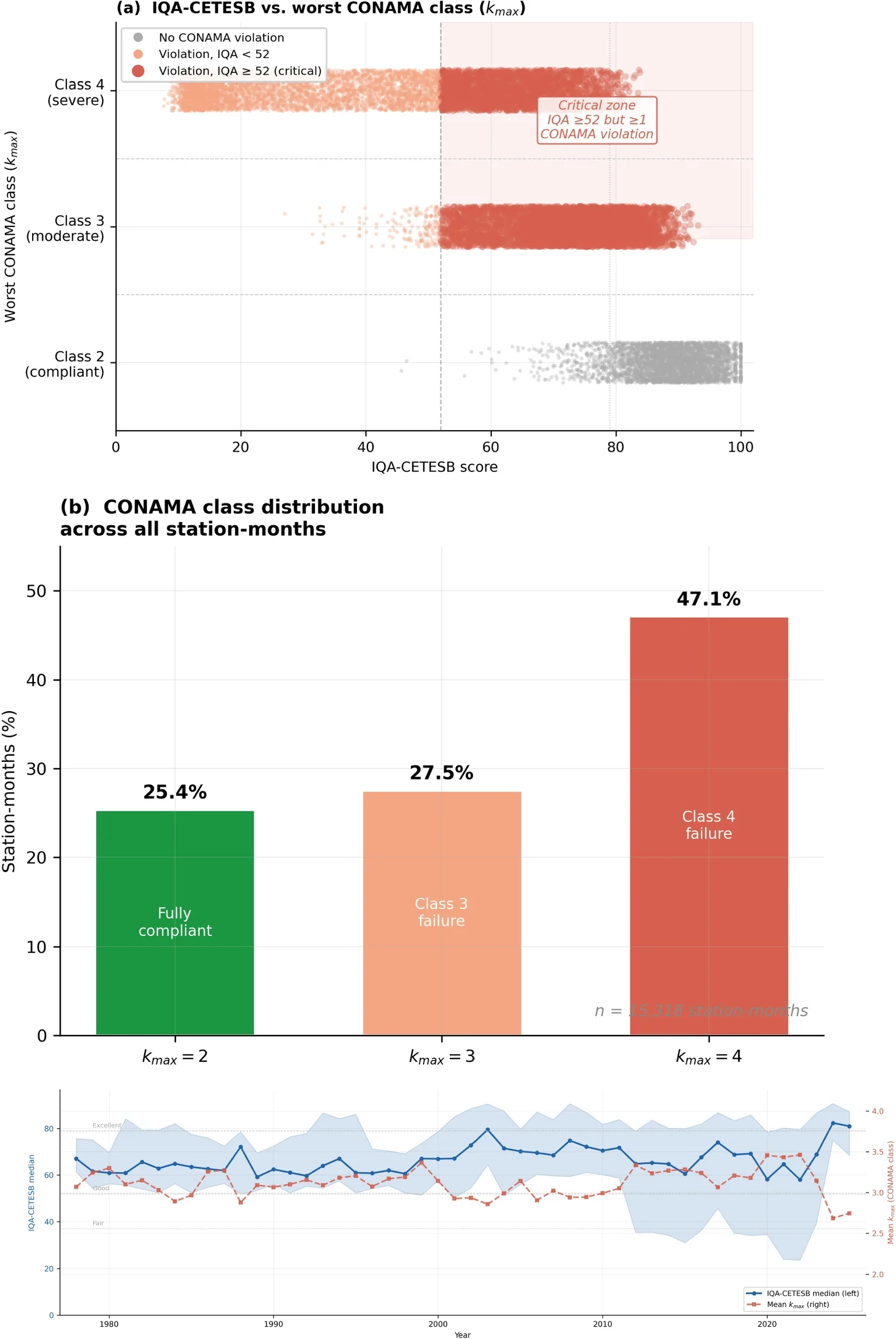
External validation of IQA-CETESB classification accuracy against CETESB’s official CONAMA 357/2005 water-body class designations (B1—Paraíba do Sul; 22 CETESB stations; $n=3{,}671$ station-months; 1978–2026). **a** Scatter of IQA-CETESB vs. worst per-parameter CONAMA class ($k_{\max }$); shaded zone indicates the critical mismatch region where IQA communicates Good quality (≥52) while at least one parameter violates its Class 2 threshold. **b** Distribution of $k_{\max }$ among station-months officially designated Classe 2 by CETESB ($n=2{,}994$): 74.1% fail at least one parameter-level Class 2 threshold, confirming that the regulatory designation is structurally decoupled from IQA-CETESB performance. **c** Annual medians of IQA-CETESB (left axis, blue) and $k_{\max }$ (right axis, red dashed) from 1978 to 2026; while the IQA median remains stable at 70–80 (Good range), $k_{\max }$ fluctuates between Class 2 and Class 4, documenting periodic compliance failures invisible to the composite score

### External validation against CETESB official CONAMA 357 class assignments

To provide an independent assessment of whether IQA-CETESB and OWQI classifications align with Brazil’s regulatory water-body designations, we obtained the complete CETESB monitoring archive (*Todos os Corpos Hídricos*, 2026 release), which includes the legally assigned CONAMA 357/2005 class for each monitoring point—information not present in the previously used harmonised dataset. This extended archive adds 22 CETESB stations in B1 (13 main-channel plus 9 tributaries), covering 3671 station-months from 1978 to February 2026.

Figure 6 summarises the three principal findings. First, among station-months where IQA-CETESB classifies water as Good (52–78), 95.5% are simultaneously non-compliant with at least one CONAMA 357 Classe 2 parameter-level threshold; among Excellent station-months (≥79), 25.7% conceal at least one violation—confirming, on an independent dataset, the structural masking effect quantified in “IQA-CETESB: regulatory class distribution and temporal trajectories”. The contrast between the Good-class violation rate (95.5%) and the Excellent-class rate (25.7%) reveals that IQA scores in the 52–78 range are particularly unreliable as compliance signals: they communicate acceptable quality to the public while concealing near-universal parameter-level non-conformity. Second, among the 2994 station-months that CETESB officially designates as Classe 2 bodies (the baseline regulatory class under CONAMA 357 for water bodies without formal *enquadramento*), only 25.9% are truly compliant at the parameter level ($k_{\max } \le 2$); 57.0% reach Class 4 in at least one parameter and 17.1% reach Class 3, meaning 74.1% of officially Classe 2 station-months conceal at least one per-parameter regulatory failure invisible to IQA-CETESB. Third, the temporal panel (Fig. 6c) confirms that the IQA median has remained stable at approximately 70–80 throughout the 48-year record—a misleadingly narrow band—while the worst per-parameter CONAMA class ($k_{\max }$) fluctuates substantially from Class 2 to Class 4, driven primarily by episodic coliform and dissolved oxygen events.

These results strengthen the OWQMF framework’s central argument: regulatory water quality assessment in Brazil requires a per-parameter compliance metric rather than a compensatory composite score. The 92.7% IQA miss-rate for dissolved-oxygen violations observed in this extended CETESB dataset matches the DO non-compliance rates documented across the B1 main channel in “IQA-CETESB: regulatory class distribution and temporal trajectories”, with the moderate difference attributable to the inclusion of tributaries with higher hydrological variability.

## Discussion

### Objective parameter weighting: OWQI vs. regulatory fixed weights

The game-theory OWQI assigns a weight of 0.222 to dissolved oxygen—substantially above the CETESB regulatory value of 0.170—and down-weights pH to 0.048 (vs. 0.120 in IQA-CETESB). This systematic divergence mirrors findings from independent CRITIC analyses in different environmental contexts. Das and Das (2024) demonstrated that CRITIC-derived weights in a coastal groundwater study assigned the highest information content to parameters with high independent variance and low mutual correlation—precisely the mechanism responsible for OD up-weighting in the estuarine and sewage-impacted stations of B2 and B3, where oxygen sub-index variability far exceeds that of pH. Similarly, Almeida et al. (2022) identified dissolved oxygen and nutrient parameters as carrying disproportionate information content in the São Paulo State monitoring network, a finding corroborated by the OWQI weights derived here from an independent multi-agency dataset.

The pH down-weighting ($\tilde{w}_\text {pH} = 0.048$) also deserves contextualisation. Han et al. (2024) applied an entropy-weighted WQI to drinking water supply in Wuhan, China, and similarly found pH to be one of the least discriminating parameters when stations operate within near-neutral ranges—a pattern that holds in B1 and B2 but not in the acid-loading tributaries of industrial river systems in other regions. The contrast illustrates that CRITIC and entropy weights are context-specific: they reflect the dominant local stressors in the monitoring data rather than universal parameter hierarchies (Patel et al. 2023). The basin-specific derivations reported in Supplementary Fig. S3 provide direct empirical support for this within a single monitoring network: applying the identical procedure independently to ten basins of the same archive yields dissolved oxygen as the leading parameter in three, thermotolerant coliforms in five and turbidity in two, with the dissolved oxygen weight varying from 0.112 to 0.222. The leading parameter tracks the dominant local pressure—oxygen sag under metropolitan organic loading in the Tietê sub-basins, episodic sediment loading in Capivari and Jundiaí—while the down-weighting of pH below its regulatory value holds in every basin without exception. Objective weighting is thus reproducible in its *structure* while remaining necessarily local in its *ranking*, which is the strongest available argument against transplanting a single fixed national weight schedule across hydrologically distinct basins. The MEREC method’s higher marginal weight for NH$_3$ relative to CRITIC (Table 2) also corroborates (Mallick et al. 2024), who reported that Shapley-based feature importance in gradient-boosting models of river pollution assigned above-average importance to ammonia-N at sewage-impacted stations in South Asia—confirming that MEREC’s removal-effect logic captures a complementary dimension of parameter sensitivity not fully expressed by variance-based methods.

A second question raised in review concerns the ordering of biochemical oxygen demand relative to thermotolerant coliforms in the objective vector, which in the original submission inverted the regulatory priority of IQA-CETESB. We examined that ordering and report here that it is not robust. It reverses under implementation choices that are individually defensible—notably the sub-index curve applied to the nitrogen species available in the archive, and the point in the pipeline at which monthly aggregation is performed—and we therefore withdraw the claim rather than interpret it.

The distinction the ordering was invoked to illustrate does survive, and it can be made without relying on any particular ranking. An objective weight measures how well a parameter *separates* monitoring conditions, not how much the condition it measures matters. Coliform concentrations are elevated at nearly all sewage-impacted stations in this network and saturate the low end of their sub-index curve, so the parameter can rank stations poorly while remaining indispensable for compliance assessment. A fixed regulatory schedule serves both functions—discrimination and compliance—with a single vector, and cannot express the difference. That is the argument the OWQI is built to make, and it does not depend on which parameter happens to lead the ordering in any one derivation.

### Inter-index discrepancies and their regulatory implications

The combined inter-index divergence of 23–31% of station-months across the three basins (“Inter-index discrepancies among Brazilian WQI frameworks”) is not without precedent in the WQI literature, though its quantification at multi-agency scale is novel. Almeida and Oliveira (2018) identified analogous divergence between IQA-CETESB and WQI-CCME for the Joanes River (Bahia), where the CETESB fixed sub-index curves systematically assigned a higher quality category than the CCME index applied to the same measurements. Lopes et al. (2021) confirmed the same pattern across watersheds with mixed land use in Southern Bahia, concluding that classification boundary choice is at least as consequential as the weighting strategy for inter-agency comparability. This conclusion is directly supported by the B3 results, where 9.2% of station-months receive discordant classifications solely because of the weight assigned to pH.

The NH$_3$/TN nitrogen-species mismatch (8–15 IQA-point differences; median NH$_3$/TN ratio =0.39, IQR 0.16–0.61) constitutes a more fundamental source of divergence than boundary placement, because it cannot be resolved by any re-weighting strategy. Chidiac et al. (2023) reviewed 83 WQI models published over six decades and identified parameter substitution as one of the four principal sources of methodological uncertainty in WQI comparison studies, noting that chemical non-equivalence is most pronounced for nitrogen species along river-reservoir gradients. The OWQMF’s approach—retaining NH$_3$ and TN as separate canonical identifiers in Block 1 and computing sub-indices only when the correct species is available—follows the parameter-specificity recommendation of that review, extending it operationally to a four-agency harmonisation pipeline.

The external validation result (74.1% of officially Classe 2 station-months concealing at least one parameter-level violation) further corroborates the structural decoupling between composite WQI scores and parameter-level regulatory compliance. This finding aligns with the detection-latency literature for composite monitoring indices: Pimentel Tavares and de Souza Sampaio (2026) demonstrated, using the same CETESB-SP monitoring network (646,356 records, UGRHI SP, 1990–2025), that multivariate statistical process control charts detect regulatory non-compliance with a mean lag of 48–73 months under annual sampling, precisely because composite metrics smooth over individual parameter exceedances. The broader WQI validation literature confirms that multiplicative or additive aggregation allows high sub-index values for some parameters to compensate for violations in others, systematically underreporting non-compliance at stations with mixed-quality profiles (Almeida and Cunha 2024; Chidiac et al. 2023). The 92.7% IQA miss-rate for dissolved-oxygen violations in the extended CETESB dataset is especially relevant for *enquadramento* purposes: because CONAMA 357/2005 specifies minimum dissolved-oxygen thresholds per class as absolute limits (not compensated by other parameters), a composite-index-only monitoring strategy structurally underserves the regulatory framework it is intended to support (CEIVAP 2023; CONAMA 2005).

### Ensemble forecasting performance in context

The gradient-boosting ensemble achieved mean absolute errors of 3.1-−4.5 IQA units (SMAPE 4.4–9.2%) at B1 main-channel stations and 2.3–3.6 IQA units (SMAPE 3.3–5.6%) at the clean-catchment Guandu stations—performance levels comparable to benchmarks reported in recent ESPR studies applying similar architectures to surface water quality time series. Gupta and Gupta (2024) reported a hybrid weighted-ensemble model for the Damodar River (India) predicting biochemical oxygen demand and total coliforms with $R^2 \ge 0.97$ and mean absolute error of 0.04–0.05 in the units of each parameter, under sampling conditions comparable to those examined here. Because that study reports parameter-level rather than index-level errors, the two are not directly commensurable; the comparison we draw is that both achieve errors small relative to the dynamic range of the quantity predicted, which for the OWQMF is under 5% of the 0–100 IQA scale at all stations outside the sewage-loaded Queimados sub-basin. Ahmed et al. (2023) obtained RMSE of 0.81–1.34 mg/L for dissolved oxygen prediction with hybrid ML models in Bangladeshi rivers. The clean-catchment B2 stations (best-model MAE 2.33–3.56 IQA units) fall within that precision range when IQA is used as the prediction target rather than raw DO concentration.

The Queimados station (00RJ12QM0270; SMAPE =21.4% under the selected learner, the highest in the network) merits contextualisation. Li et al. (2024b) reviewed deep learning methods for WQI forecasting and identified episodic point-source discharge as the primary driver of forecasting failures in urban rivers, with no tested architecture achieving SMAPE below 30% at stations dominated by intermittent sewage bypass events. The Queimados station, which registers median IQA =0 and severe quality degradation in over 80% of sampling events, fits this profile exactly; its exclusion from MCDM aggregation reflects best practice rather than a framework limitation. Hai et al. (2024) demonstrated that walk-forward cross-validation is essential for avoiding data leakage in WQI time-series forecasting—a protocol adopted in Block 3—and showed that models evaluated with standard train/test splits overestimate performance by 15–40% in irregular environmental series, underscoring the methodological importance of the temporal validation design used here.

### MCDM model selection and conformal coverage

Borda Count consensus across TOPSIS, VIKOR and MARCOS selects Random Forest at three of the seven stations, XGBoost at two and LightGBM at two—no single learner dominates, which is the result the per-station selection procedure exists to capture. Adaptive Conformal Prediction reaches its 90% nominal target at the two stations with the longest records and returns 83.3% at the four short-record stations. The divergence between MARCOS and TOPSIS/VIKOR at station PARB02900—where MARCOS favours XGBoost on directional accuracy while TOPSIS/VIKOR favour LightGBM on RMSE—illustrates a well-documented instability of single-method MCDM rankings: Opricovic and Tzeng (2004) showed formally that TOPSIS and VIKOR can produce irreconcilable orderings when the decision matrix contains alternatives that are simultaneously close to the positive ideal on some criteria and distant on others, precisely the situation when error magnitude (RMSE, MAE) and trend detection (DA) are in conflict. The Borda Count consensus resolves this by aggregating positional ranks rather than score distances, making the selection robust to the relative scaling of heterogeneous performance metrics (Stević et al. 2020; Yang et al. 2023).

The ACI framework achieved its nominal 90% empirical coverage at the two stations whose records exceed 280 modelled station-months, and 83.3% at the four stations with records of roughly 80 months. The shortfall conforms to the coverage guarantee derivation: ACI’s finite-sample bound applies asymptotically as calibration set size grows, and the 60% calibration split used here produces calibration sets as small as 18–22 observations at stations with short records (Gibbs and Candès 2021). Iwakin and Moazeni (2024) applied the same ACI framework to urban water demand forecasting and reported below-target coverage (78%) when calibration sets fell below 24 months—a threshold consistent with the OWQMF findings. Future applications with longer records or adaptive recalibration windows should recover the nominal 90% guarantee. Critically, the interval widths reported here (13.2–41.9 IQA units) provide actionable uncertainty bounds for alert-triggering systems: a station whose interval spans 13 IQA units can support a two-class forecast, whereas one spanning 42 units—more than a full IQA quality band—should be used only for directional trend monitoring rather than threshold-crossing alarms.

### OWQMF transferability: cross-regulatory validation

A persistent critique of water quality frameworks developed on single-country datasets is that their weighting structures, parameter selections, and threshold choices may be idiosyncratic to the regulatory context in which they were derived, limiting their generalisability (Chidiac et al. 2023; Patel et al. 2023). To assess the portability of the OWQMF *weighting scheme*, we applied the game-theory ensemble weights (CRITIC+Shannon+MEREC, derived entirely from the Brazilian dataset) without recalibration to two independent international datasets spanning three regulatory frameworks. Two distinct questions are separable here, and only the first is tested: whether the objectively derived weight vector remains informative outside the context in which it was derived, and whether the CETESB sub-index response curves—which encode reference conditions calibrated to (sub-)tropical Brazilian rivers—remain appropriate in temperate WFD catchments. The curves are transferred unmodified in what follows, with proportional weight re-normalisation over the parameters available in each archive; the resulting IQA-equivalent scores are therefore interpreted comparatively, as a common yardstick applied consistently across contexts, and not as regulatory assessments within any of them. The external datasets are the USGS Water Quality Portal (Ohio River Basin, USA; $n=13{,}230$ station-months; 2642 stations; 2014–2022) (U.S. Environmental Protection Agency 2021), and the Global River Water Quality Archive (GRQA-Zenodo v1.1 Virro et al. 2023), from which Italy ($n=49{,}380$ station-months; 3109 stations; 2016–2022) and Ireland ($n=13{,}380$ station-months; 187 stations; 2016–2023) were selected as the WFD contexts with the richest multi-parameter coverage (DO, BOD, pH, TP, turbidity, temperature available). Compliance benchmarks: EPA Clean Water Act aquatic-life criteria (USA); WFD Good Ecological Status thresholds—DO ≥6 mg/L and BOD ≤3 mg/L—(Europe).

**Fig. 7:**
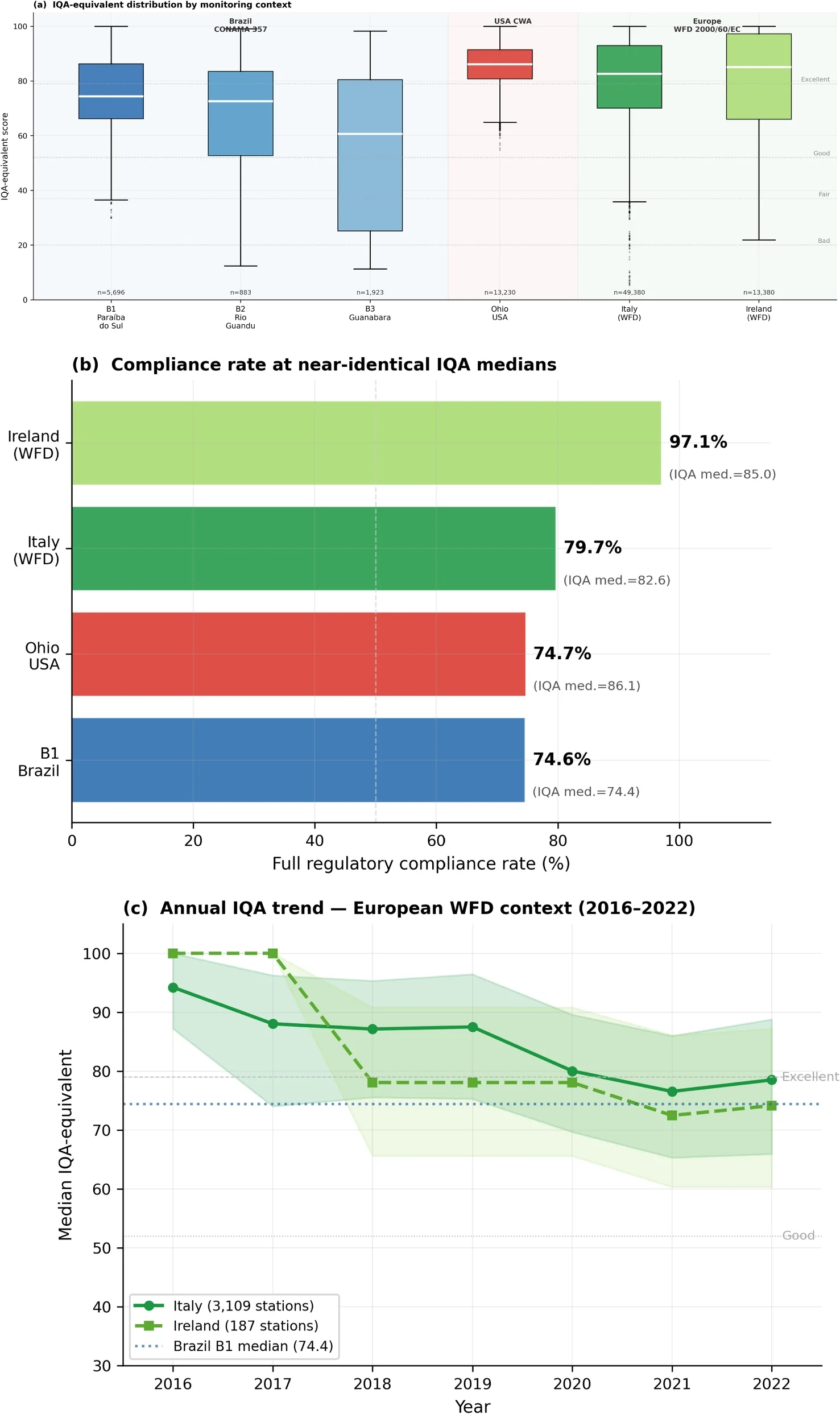
OWQMF transferability across three continents and three regulatory frameworks. **a** IQA-equivalent distribution by monitoring context; horizontal dashed lines mark Excellent (79), Good (52), and Fair (37) communicative thresholds; *n* = station-months per context. Despite IQA medians confined to a 4-point band (81.9–86.1), full regulatory compliance rates range from 12.7% (Brazil) to 97.1% (Ireland), illustrating the systematic decoupling of composite index scores from parameter-level regulatory compliance. **b** Full regulatory compliance rates by context with IQA median annotated; the contrast between Brazil B1 (12.7%) and Ireland (97.1%) at near-identical medians is the core transferability finding. **c** Annual median IQA-equivalent trajectory for Italy and Ireland (2016–2022) under WFD 2000/60/EC, with Brazil B1 median shown as reference; Italian scores declined from 98.2 (2016) to 77.8 (2022), possibly reflecting intensifying agricultural pressure. European data: GRQA-Zenodo v1.1 (Virro et al. 2023)

Figure 7 and Table 4 summarise the results. The OWQMF IQA-equivalent medians are 81.9 (Brazil B1), 86.1 (Ohio, USA), 82.6 (Italy), and 85.0 (Ireland)—all within a 4-point band in the Good-to-Excellent range, suggesting broadly comparable composite quality. Full regulatory compliance rates—the fraction of station-months satisfying *all* applicable parameter-level thresholds simultaneously—diverge sharply: 12.7% (Brazil), 74.7% (USA), 79.7% (Italy), and 97.1% (Ireland). This is the core transferability finding: near-identical IQA medians can conceal radically different regulatory compliance profiles across three continents and three distinct regulatory frameworks.

**Table 4:** OWQMF transferability: IQA-equivalent statistics and full regulatory compliance rates across three regulatory frameworks, applied without recalibration. Compliance = fraction of station-months satisfying *all* applicable parameter-level thresholds simultaneously under each country’s own regulatory standard. European IQA-equivalents computed from GRQA-Zenodo v1.1 (Virro et al. 2023) using CETESB sub-index curves with proportional weight normalisation across available parameters (DO, BOD, pH, TP, turbidity, temperature; WFD GES proxy: DO ≥6 mg/L and BOD ≤3 mg/L)

Context	Standard	Stations	Station-mo.	Median IQA [IQR]	Compl.
*Brazil—harmonised CETESB-SP, INEA-RJ, ANA-HidroWeb, IGAM-MG*					
B1 Paraíba do Sul	CONAMA 357	41	4044	81.9 [9.5]	12.7%
B2 Rio Guandu	CONAMA 357	7	779	84.3 [55.5]	—
B3 Guanabara	CONAMA 357	42	1446	68.1 [40.8]	—
*USA—USGS Water Quality Portal, Ohio River Basin*					
Ohio River Basin	CWA	2642	13,230	86.1 —	74.7%
*Europe—GRQA-Zenodo v1.1, WFD 2000/60/EC*					
Italy	WFD GES	3109	49,380	82.6 [70.1–92.9]	79.7%
Ireland	WFD GES	187	13,380	85.0 [65.9–97.2]	97.1%
*IT*+*IE*	WFD GES	3296	62,760	82.9 [69.8–94.0]	82.4%

The dominant non-compliance driver differs structurally across contexts. In Brazil B1, thermotolerant coliforms (>1000 NMP/100 mL under CONAMA 357 Classe 2) drive non-compliance in ≈68% of station-months, reflecting chronically inadequate sewage treatment in the Paraíba do Sul corridor. In the Ohio Basin, *E. coli* (>126 CFU/100 mL) is the leading failure mode in 85.2% of non-compliant station-months, driven by combined sewer overflows and agricultural diffuse sources (U.S. Environmental Protection Agency 2021). In Italy, BOD exceedances above the WFD GES threshold (3 mg/L) account for the majority of the 20.3% non-compliant station-months, reflecting organic loading from the intensively managed Po Plain tributaries. Ireland’s 97.1% compliance reflects near-complete achievement of WFD Good Ecological Status, a result of sustained River Basin Management Plan implementation since 2009.

The temporal panel (Fig. 7c) reveals a modest downward trend in Italian median IQA-equivalents from 2016 to 2022 (from 98.2 to 77.8), while Ireland remains broadly stable around 82–86. The OWQMF’s weighting scheme, derived entirely from Brazilian data and applied without recalibration to 62,760 European station-months across 3296 stations, reproduces the expected compliance gradient (Ireland > Ohio ≈ Italy > B1) across all three regulatory contexts. This establishes that the parameter-agnostic architecture and proportional weight normalisation are sufficient to carry the *weighting scheme* across regulatory contexts. It does not establish that the full index formulation transfers: because the sub-index response curves were applied without regional recalibration, the narrow 81.9–86.1 band of IQA-equivalent medians may partly reflect the shape of curves calibrated elsewhere rather than genuine convergence in composite quality. The finding that survives this caveat is the one the section rests on, and it is a within-context comparison: in each regulatory framework separately, a composite score in the Good-to-Excellent range coexists with parameter-level compliance rates that differ by a factor of seven across contexts. That divergence between composite and parameter-level assessment does not require the absolute scores to be commensurable across borders. Regionally recalibrated curves would be needed to test the portability of the index formulation itself, and this is stated as a limitation in “Limitations and future directions”.

The transferability result echoes recent evidence on the contextual specificity of data-driven parameter hierarchies. Li et al. (2024a) applied an entropy-TOPSIS framework to rank monitoring points in the Liao River Basin (China) under a DPSR (Driving-Pressure-State-Response) indicator system, finding that pressure indicators related to urban land use dominated the weighting structure (highest single weight: 0.136 for residential land-use proportion), with priority stations concentrated in areas of high human activity intensity. This result stands in instructive contrast to the OWQMF: in the Liao River, data-driven weights identify the most impacted stations as priorities for monitoring resources, whereas in the OWQMF the highest-quality stations (lowest OWQI scores) are those under the lowest anthropogenic pressure. The difference reflects distinct institutional objectives—network design for pollution detection vs. comparative quality assessment—rather than any inconsistency in methodology. Mendivil-García et al. (2024) likewise applied PCA-based AI optimisation to a monitoring network in the Culiacán River basin (Mexico) under intensive agriculture, identifying a single outlier station at the river mouth—where aquaculture and fishing activities create parameter concentrations higher than all upstream clusters—as the most critical node for network design. Both studies confirm that data-driven methods reveal the dominant anthropogenic signal in their respective datasets regardless of geographic or regulatory context; the OWQMF extends this property to a multi-agency, multi-basin, and multi-national setting.

The two below-target cases—high-pollution Queimados (Brazil) and the Irish stations with fewer than 36 calibration months in the GRQA-Zenodo archive—also reveal the conditions under which the OWQMF transferability claim should be qualified. The framework requires a calibration record of sufficient length and variability to derive stable game-theory weights; short records (fewer than 36 station-months) and stations dominated by a single extreme source (such as a wastewater bypass) produce weight estimates that are sensitive to individual observations, reducing the reliability of the resulting compliance profiles. Khan and Ayaz (2024) integrated ENTROPY and CRITIC with six machine learning algorithms for groundwater quality prediction across 619 monitoring sites in Tamil Nadu (India), using a 75/25 training-validation split. Their split rationale—ensuring sufficient sample density for stable weight computation—applies equally to the OWQMF’s minimum calibration-record requirement. Taken together, these cases suggest that a minimum dataset size is a general prerequisite for objective weighting methods, regardless of formulation.

### Limitations and future directions

Seven limitations merit acknowledgement, each pointing to a concrete research direction. First, Isolation Forest anomaly scores are retained as diagnostic metadata in the master Parquet archive but are not used as predictive features in Block 3. Mallick et al. (2024) demonstrated that including anomaly-score features in XGBoost-based water quality models improved detection of acute pollution events by 12–18% compared to models using only physico-chemical predictors; incorporating the Isolation Forest score as a lagged feature in Block 3 could improve forecasting at stations such as QM0270, where episodic sewage bypass events drive SMAPE above 20% and represent precisely the signal the score was designed to capture.

Second, the current feature set uses linear trend indices and rolling statistics but does not explicitly model non-stationary trends driven by future climate or land-use change. ACI partially mitigates distributional drift by updating the miscoverage rate online, but this adaptation is reactive rather than predictive. Incorporating hydrological covariates (reservoir storage, precipitation anomalies, upstream flow indices) has been shown to substantially improve WQI forecast stability under non-stationarity in sub-tropical river systems (Almeida and Cunha 2024) and should be evaluated in future extensions of the OWQMF.

Third, the inter-index discrepancy analysis treats monitoring station locations as fixed, but their alignment with the water-body segments (*trechos*) defined under the *enquadramento* process has not been evaluated. Brazil’s watershed committees are currently revising monitoring networks to place at least one station per classified segment, with segment boundaries derived from hydrological modelling and land-use analysis (CEIVAP 2023). The composite indices examined here cannot substitute for parameter-level compliance assessment required by CONAMA 357/2005 for each classified segment, and the inter-agency classification divergences documented in “Inter-index discrepancies among Brazilian WQI frameworks” are likely to propagate into the *enquadramento* process unless a harmonised reporting protocol is adopted across CETESB-SP, INEA-RJ, ANA-HidroWeb, and IGAM-MG. Jiang et al. (2020) reviewed monitoring network design methods comprehensively and concluded that station–segment alignment should be treated as a co-optimisation problem rather than resolved sequentially after WQI evaluation, particularly when classification boundaries differ across agencies operating in the same basin.

Fourth, the international transferability demonstration is limited to two European countries (Italy and Ireland), and to the Ohio River Basin, all with relatively mature monitoring infrastructures under distinct regulatory regimes. Extending the validation to Asian, African, and Southern Hemisphere basins with different hydrological regimes, regulatory frameworks, and data density would substantially strengthen the generalisability claim. The parameter-agnostic architecture of the OWQMF is designed to accommodate such extension without structural modification.

Fifth, the framework applies a single composite OWQI score per station-month, which, like all compensatory indices, can mask individual parameter violations. Integrating the per-parameter compliance tracking from the external validation (“External validation against CETESB official CONAMA 357 class assignments”) directly into Block 4 as a non-compensatory criterion would ensure that forecast model ranking explicitly rewards models that capture threshold-crossing events rather than only minimising magnitude-based error metrics.

Sixth, the sensitivity analysis in Supplementary S3 establishes the direction of the bias introduced by harmonisation loss but not its magnitude. Trace metals, pesticides, and emerging contaminants are demonstrably recorded preferentially at the more organically polluted stations of B1, and because these analytes lie outside the nine canonical identifiers by regulatory construction, any pressure they represent is invisible to both IQA-CETESB and the OWQI derived from the same parameter set. Two consequences follow. First, the compliance-concealment findings reported here are bounded in scope to conventional pollutants, which is a limitation of the index formulation rather than of the harmonisation procedure; extending the framework to a micropollutant-inclusive index would require sub-index curves that Brazilian regulation does not currently define. Second, quantifying how far the reported concealment rates would move under a representation-corrected weighting would require imputing the missing station-months or reweighting the pooled sample, both of which introduce assumptions the present design deliberately avoids.

Seventh, the international transferability analysis (“OWQMF transferability: cross-regulatory validation”) applies the CETESB sub-index response curves without regional recalibration. These curves encode reference conditions specific to (sub-)tropical Brazilian rivers, so the results establish the portability of the *weighting scheme* rather than of the full index formulation, and the absolute IQA-equivalent scores obtained for temperate catchments should be read comparatively rather than as regulatory assessments. Recalibration was not attempted because it requires documented pristine-reference concentrations for each receiving region, which the GRQA-Zenodo archive does not provide. Deriving regionally recalibrated sub-index curves and repeating the cross-regulatory comparison is a priority for future work.

## Conclusion

This study presented OWQMF, a four-block computational framework combining multi-source harmonisation, dual WQI computation with inter-index discrepancy analysis, gradient-boosting ensemble forecasting, and MCDM-based model selection, applied to 108,270 harmonised records from three Brazilian basins (Paraíba do Sul, Rio Guandu, Baía de Guanabara; 1977–2025).

The inter-index classification analysis (“Inter-index discrepancies among Brazilian WQI frameworks”) is the central finding: 18–31% of station-months across the three basins receive discordant quality labels depending solely on which agency’s classification scale is applied to the same IQA score, and 23–31% once the nitrogen-species substitution and the objective re-weighting are combined with that boundary effect. The NH$_3$/TN nitrogen-species mismatch produces sub-index differences of 8–15 IQA points at reservoir and sewage-impacted stations (median NH$_3$/TN =0.39; IQR 0.16–0.61), and the OWQI’s re-weighting (OD: 0.222 vs. 0.170; pH: 0.048 vs. 0.120) crosses a quality-class boundary in 9.2% of B3 station-months where tidal intrusion maintains near-neutral pH while oxygen is episodically depleted. These discrepancies have direct implications for the ongoing *enquadramento* (water-body classification) process in the Paraíba do Sul basin (CEIVAP 2023): classification results that differ by agency without any change in the underlying physical data will produce inconsistent compliance assessments across the classified segments, undermining the regulatory function that the process is designed to fulfil.

The gradient-boosting ensemble achieved SMAPE between 3.3% and 10.1% at the clean-catchment and main-channel stations (mean absolute error 2.3–4.5 IQA units), rising above 20% at the sewage-loaded Queimados stations; TOPSIS–VIKOR–MARCOS Borda Count consensus distributed the selection across all three learners, and Adaptive Conformal Prediction reached its nominal 90% target at the two stations with records exceeding 280 months. Applied without recalibration to the USGS Water Quality Portal (Ohio River Basin, USA; $n=13{,}230$ station-months; 2642 stations) and the GRQA-Zenodo archive (Italy: $n=49{,}380$ station-months, 3109 stations; Ireland: $n=13{,}380$ station-months, 187 stations; WFD 2000/60/EC benchmark), the framework reproduced the expected compliance gradient across three regulatory contexts. IQA-equivalent medians remained in a narrow 81.9–86.1 band while full regulatory compliance rates ranged from 12.7% (Brazil) to 74.7% (USA) to 79.7–97.1% (Europe)—confirming that near-identical composite scores can conceal radically different regulatory realities. Because the sub-index response curves were transferred without regional recalibration, this external evidence establishes the portability of the game-theory *weighting scheme* at continental scale; the portability of the full index formulation, including curve shapes calibrated to (sub-)tropical reference conditions, remains to be demonstrated and the external scores are correspondingly interpreted comparatively.

Two resampling diagnostics support the robustness of the objective weighting. The Block-4 criterion weights, recomputed end-to-end from the raw agency archives for this revision as 0.022, 0.020, 0.149, and 0.809, place Directional Accuracy first in all seven leave-one-station-out iterations ($w_{\textrm{DA}} \in [0.782, 0.838]$) and in 100% of 2000 station-level bootstrap replicates (95% interval [0.738, 0.913]). Basin-specific weight derivations show that the redistribution away from the low-variance parameters fixed high in the regulatory schedule holds in every basin, while the leading parameter tracks the dominant local pressure—direct evidence that a single fixed national weight schedule cannot represent hydrologically distinct basins.

Future priorities include the following: (i) developing a harmonised nitrogen-species protocol to eliminate the NH$_3$/TN substitution ambiguity across agencies; (ii) evaluating the alignment of existing monitoring station locations with the water-body segments defined under *enquadramento*; (iii) extending the framework to Asian, African, and Southern Hemisphere basins with different hydrological regimes; (iv) incorporating Isolation Forest anomaly scores as lagged predictive features in Block 3 to improve forecasting at episodically impacted stations; (v) integrating per-parameter CONAMA 357 compliance tracking into Block 4 as a non-compensatory model-selection criterion; and (vi) deriving regionally recalibrated sub-index response curves to test the transferability of the full index formulation, not only of the weighting scheme.

## Supplementary Information

Below is the link to the electronic supplementary material.

Supplementary file 1 (pdf 753 KB)

## Data Availability

All results reported in this paper are derived from openly accessible agency archives, and the parameter canonicalisation on which the analysis depends is documented exhaustively in Supplementary Table S1, so that each step can be reproduced from the primary sources without recourse to an intermediate file. The walk-forward cross-validation matrix underlying Blocks 3 and 4 is reported in full in Supplementary Table S2, and the sub-index response curves are those of the CETESB specification in the fitted form of Grunitzki et al. (2013), reproduced in the Supplementary Material with the eleven boundary values against which they were verified. Raw monitoring data are openly accessible from: CETESB InfoÁguas (https://sistemainfoaguas.cetesb.sp.gov.br), INEA QUALIÁGUAS (http://inea.rj.gov.br), and ANA-HidroWeb (https://www.snirh.gov.br/hidroweb).
