# Supplementary material for: Data-driven optimisation of multi-agency river water quality monitoring networks under high anthropogenic pressure
Source: Environ Sci Pollut Res Int. 2026 Sep 4;33(28):14446–69. doi: 10.1007/s11356-026-38166-0 (PMC13582203; doi:10.1007/s11356-026-38166-0)
Supplement: Supplementary file 1 [file 11356_2026_38166_MOESM1_ESM.pdf]

Supplementary Material

Data-Driven Optimisation of Multi-Agency River Water Quality Monitoring Networks Under High Anthropogenic Pressure

Manuscript ID: ESPR-D-26-05463 — Revision 1

1 S1. Parameter nomenclature correspondence

Table S1 maps every raw parameter string encountered in the four agency archives onto the nine canonical IQA identifiers. The mapping is generated programmatically from the archives themselves and is reproduced by the script accompanying this revision, so that the canonicalisation step is auditable rather than asserted.

Two features of the table bear on the analysis. First, the same analyte is recorded under as many as eight distinct strings, differing in unit suffix, whitespace, capitalisation and language; turbidity alone appears as `Turbidez`, `Turbidez (UNT)`, `Turbidez (uT)` and `24_Turbidez_ntu`. Second, ANA-HidroWeb supplies only three of the nine identifiers, so that archive cannot support an IQA computation at any of its stations and enters the study as a supplementary reference only.

Table S1: Correspondence between raw parameter strings and the nine canonical IQA identifiers, by agency. Dashes indicate that the agency does not report the parameter. *Total* is the number of distinct raw strings mapped onto each identifier.

Canonical identifier	Total	CETESB-SP / IGAM-MG	INEA-RJ / ANA-HidroWeb
Dissolved oxygen	4	Oxigênio Dissolvido	OD (mg/L); OD (mg/L) / —
Therm. coliforms	8	Coliformes Termotolerantes; Escherichia coli**	Coliformes Termotolerantes (NMP/100 mL); (NMP/100mL); Escherichia coli (NMP/100 mL) / 10_Escherichiacoli_ufc_100ml
pH	3	pH	pH / —
$BOD_{5,20}$	3	DBO (5, 20); DBO — Demanda Bioquímica de Oxigênio	DBO (mg/L) / —
Nitrogen (NH_3-N)	5	Nitrogênio Amoniacal	Nitrogênio Amoniacal Total (mg/L); Nitrogênio amoniacal total (mg/L) / 14_Nitrogenio_Amoniacal_mgl
Total phosphorus	3	Fósforo Total	Fósforo Total (mg/L) / —
Water temperature	3	Temperatura da Água	Temperatura da Água (°C) / —
Turbidity	5	Turbidez	Turbidez (UNT); Turbidez (uT) / 24_Turbidez_ntu
Total solids	4	Sólido Total; Sólidos Totais	Sólidos Totais (mg/L); Sólidos totais (mg/L) / —

2 S2. Walk-forward cross-validation results

Table S2 reports the walk-forward cross-validation metrics for the three learners at every station entering Block 3. The series forecast is monthly IQA-CETESB computed with the fixed regulatory weight schedule and the sub-index response curves specified in the Appendix E of CETESB,

in the fitted form of Grunitzki et al. (2013, XX SBRH); the fitted curves reproduce all eleven boundary values given in the official specification. The cross-validation is expanding-window with $n_{\text{test}} = 10$ held-out months, one-step-ahead, refitting at each step.

Two of the nine stations originally entering Block 3 are excluded here on data-availability grounds, both verifiable from the archives. Station 02RJ10GN0064 (Baía de Guanabara) reports no total-solids measurement at any date, and the IQA specification is explicit that the absence of any one of the nine variables makes the computation unfeasible. Station 00RJ12GM0180 (Rio Guandu-Mirim) yields 19 valid station-months, which is insufficient for a feature set containing a twelve-month autoregressive lag plus ten held-out months. Seven stations therefore enter Blocks 3 and 4.

Table S2: Walk-forward cross-validation metrics by station and learner. RMSE and MAE in IQA units; SMAPE and DA (directional accuracy) in per cent. n is the number of modelled station-months after feature construction. Bold marks the learner selected by the Block-4 consensus. INEA-RJ station codes are abbreviated from their 00RJ12 and 02RJ10 prefixes.

**Basin**	**Station**	n	**Learner**	**RMSE** (IQA)	**MAE** (IQA)	**SMAPE** (%)	**DA** (%)
B1	PARB02600	327	XGBoost	5.57	4.67	9.55	77.8
			LightGBM	6.17	4.93	10.07	55.6
			Random Forest	**5.36**	**4.51**	**9.18**	**77.8**
B1	PARB02900	326	XGBoost	**4.77**	**4.33**	**7.34**	**88.9**
			LightGBM	5.40	4.93	8.41	77.8
			Random Forest	4.96	4.53	7.77	77.8
B1	PARB02100	289	XGBoost	4.58	3.49	4.96	77.8
			LightGBM	4.85	3.88	5.38	66.7
			Random Forest	**3.87**	**3.10**	**4.39**	**77.8**
B2	GN0200	76	XGBoost	4.36	3.84	5.97	44.4
			LightGBM	**4.21**	**3.56**	**5.55**	**55.6**
			Random Forest	4.14	3.58	5.58	55.6
B2	GN0201	80	XGBoost	3.07	2.75	3.89	44.4
			LightGBM	3.41	2.39	3.42	66.7
			Random Forest	**2.93**	**2.33**	**3.25**	**66.7**
B3	QM0270	77	XGBoost	7.53	6.60	22.65	33.3
			LightGBM	**7.46**	**6.18**	**21.42**	**44.4**
			Random Forest	6.96	5.94	20.21	33.3
B3	QM0271	76	XGBoost	**5.14**	**3.84**	**16.98**	**44.4**
			LightGBM	6.20	5.32	24.53	44.4
			Random Forest	5.49	3.90	16.95	44.4

3 S3. Sensitivity of water quality indices to harmonisation loss

S3.1 Rationale and scope

Reviewer 1 observed that parameter-name canonicalisation retains only a fraction of the raw archive, and raised a specific mechanism: that trace-metal- and micropollutant-intensive stations, which tend to be the most industrially impacted, may be disproportionately affected, so that the loss would be informative rather than neutral.

This analysis is scoped deliberately. It is computed on the 22 CETESB-SP stations of basin B1 (Paraíba do Sul main channel and UGRHI-2 tributaries; 126,472 raw records; 1978–2026), which are the stations for which the complete raw archive, including every analyte outside the nine canonical IQA identifiers, is available at record level. The agency-specific extracts for

INEA-RJ and ANA-HidroWeb are distributed as pre-filtered parameter sets and therefore cannot support a loss analysis of this kind: the excluded records are not present to be counted. B1 is nonetheless the appropriate test bed, since it is the basin with the lowest harmonisation rate and the one the reviewer's comment addresses.

Two quantities are defined per station s. The *harmonisation completeness* h_s is the share of raw records at s mapping to one of the nine canonical IQA identifiers, and the *micropollutant intensity* m_s is the share of raw records belonging to CETESB's trace-metal, volatile and semi-volatile organic, PAH, pesticide, PFAS, dioxin and rare-earth analyte classes. Across the 22 stations, h_s has median 24.9% (range 17.7–37.9%).

S3.2 The reviewer's mechanism is confirmed

Harmonisation completeness and micropollutant intensity are strongly inversely related (Spearman $\rho = -0.816$, $p < 10^{-5}$; bootstrap 95% CI $[-0.94, -0.57]$, 10,000 resamples; Figure S1a). Stations that harmonise poorly do so because a larger share of their monitoring effort is directed at analytes that lie outside the IQA parameter set by regulatory construction. The reviewer's proposed mechanism is therefore not merely plausible: it is the dominant one.

S3.3 Consequences, and the direction of the resulting bias

We then tested whether stations differing in h_s differ in water quality, splitting at the median and comparing station medians for each of the nine IQA parameters (Table S3). Two of the eight testable parameters differ significantly, and both survive Bonferroni correction at $\alpha = 0.05/8$: biochemical oxygen demand ($p = 0.0027$, $r = 0.58$) and ammonia-N ($p = 0.0010$, $r = 0.69$). In both cases the low-harmonisation group is the more polluted one, with median BOD of 3.0 against 2.0 mg/L and median ammonia-N of 0.50 against 0.12 mg/L. Dissolved oxygen, coliforms, total phosphorus, turbidity, total solids and pH do not differ.

Low-harmonisation stations are additionally under-represented in the index computation. They yield a median of 72 station-months with all nine parameters simultaneously present, against 245 for the high-harmonisation group ($p = 0.015$), and a smaller share of their station-months clears the nine-parameter validity gate (80.0% against 90.7%, $p = 0.0039$; overall 3,211 of 3,666 station-months, 87.6%). Harmonisation completeness and the number of IQA-computable station-months are positively associated (Spearman $\rho = +0.477$, $p = 0.025$; Figure S1c).

We therefore do not claim that harmonisation loss is non-informative. It is informative, in the direction the reviewer anticipated. What the analysis establishes instead is the sign of the resulting bias, and it is the conservative sign. The excluded analytes are metals and organic micropollutants, which are not IQA inputs under CONAMA 357/2005; their removal cannot alter the IQA value computed at any station, because they never entered the computation. What the loss does is reduce the weight carried in the pooled analysis by the dirtier stations. Since the central finding of this study is that composite indices conceal parameter-level non-compliance, and since the stations lost to attrition are those with higher organic and sewage loading, the concealment rates reported in Section 4.6 are lower bounds. Correcting the imbalance would move them upward, not downward.

S3.4 Limitation

This analysis establishes the direction of the bias, not its magnitude. Quantifying how much the reported concealment rates would move under a representation-corrected weighting would require either imputing the missing station-months or reweighting the pooled sample, both of which introduce assumptions the present design avoids. The analysis is also confined to CETESB-SP records, for the reason given in S3.1; whether INEA-RJ and ANA-HidroWeb monitoring exhibits the same coupling between micropollutant effort and canonical-parameter attrition cannot be determined from the extracts available.

Table S3: Comparison of station-median water quality between high- and low-harmonisation stations, 22 CETESB-SP stations of basin B1, split at the median harmonisation completeness ($h_s = 24.9\%$; $n = 11$ per group). Two-sided Mann–Whitney U; r is the rank-biserial effect size. Bold indicates significance after Bonferroni correction across the eight parameters ($\alpha = 0.00625$).

**Parameter**	**Unit**	**High**	**Low**	U	p	r
Dissolved oxygen	mg/L	5.90	6.50	55	0.767	0.07
**BOD$_{5,20}$**	mg/L	**2.00**	**3.00**	**19**	**0.0027**	**0.58**
Therm. coliforms	NMP/100 mL	4950	1910	75	0.358	0.20
**Ammonia-N**	mg/L	**0.12**	**0.50**	**11**	**0.0010**	**0.69**
Total phosphorus	mg/L	0.040	0.050	53	0.643	0.11
Turbidity	NTU	15.5	20.0	56	0.793	0.06
Total solids	mg/L	62.0	51.0	74	0.382	0.19
pH	—	6.80	6.75	66	0.715	0.08
Representation in the index computation						
IQA station-months	count	245	72	98	0.015	—
Nine-parameter gate	%	90.7	80.0	105	0.0039	—

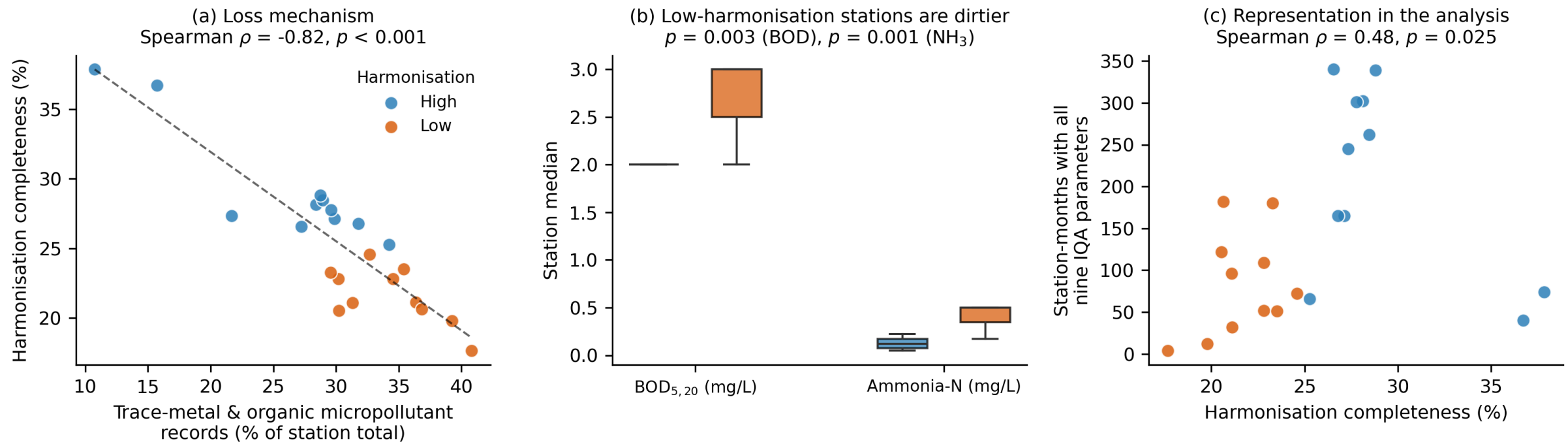

Figure S1: Harmonisation loss in basin B1 (22 CETESB-SP stations). (a) Harmonisation completeness against trace-metal and organic micropollutant intensity; the loss is driven by monitoring effort directed at non-IQA analytes. (b) Station-median BOD and ammonia-N by harmonisation group; low-harmonisation stations are the more polluted. (c) Harmonisation completeness against the number of station-months with all nine IQA parameters present; poorly harmonising stations are under-represented in the index computation.

4 S4. Stability of Block-4 CRITIC criterion weights

S4.1 Rationale and design

Reviewer 1 observed that the four MCDM criterion weights (RMSE, MAE, SMAPE, DA) are derived from a thin station–model matrix ($n = 21$ rows: seven stations × three models), that the dominance of Directional Accuracy drives model selection at every station, and that a stability check was needed before that dominance could be trusted. We agree, and report it here on the metric matrix itself.

Recomputing the weights directly from the walk-forward cross-validation matrix for this revision gives $w_{\mathrm{RMSE}} = 0.0222$, $w_{\mathrm{MAE}} = 0.0200$, $w_{\mathrm{SMAPE}} = 0.1486$ and $w_{\mathrm{DA}} = 0.8093$. These supersede the values reported in the original submission (0.205, 0.199, 0.196, 0.400). The correction increases the concentration of weight on Directional Accuracy rather than reducing it, and it does not propagate: the model selected by the TOPSIS–VIKOR–MARCOS Borda consensus is identical at all nine stations under both weight vectors.

Two diagnostics are applied. Leave-one-station-out (LOSO) removes each of the seven stations in turn and recomputes all four weights from the remaining six; the non-parametric bootstrap resamples stations with replacement (2,000 replicates). Both operate at the *station* level rather than the row level, because the three model rows belonging to one station are not independent and row-level resampling would understate uncertainty.

Table S4: Leave-one-station-out stability of the Block-4 CRITIC criterion weights, computed on the 21-row station–model matrix. Each row recomputes all four weights with the indicated station removed. Directional Accuracy retains the highest weight in every iteration.

**Station removed**	w_{RMSE}	w_{MAE}	w_{SMAPE}	w_{DA}
00RJ12GN0200	0.0225	0.0203	0.1552	**0.8020**
00RJ12GN0201	0.0218	0.0197	0.1465	**0.8120**
00RJ12QM0270	0.0231	0.0209	0.1340	**0.8220**
00RJ12QM0271	0.0226	0.0204	0.1350	**0.8220**
PARB02100	0.0215	0.0193	0.1592	**0.8000**
PARB02600	0.0219	0.0197	0.1764	**0.7820**
PARB02900	0.0212	0.0190	0.1223	**0.8375**
Minimum	0.0212	0.0190	0.1223	0.7820
Maximum	0.0231	0.0209	0.1764	0.8375
Mean	0.0221	0.0199	0.1469	0.8077

Table S5: Bootstrap distribution of the Block-4 CRITIC criterion weights (2,000 resamples of stations with replacement, 21-row matrix). CI: 95% percentile interval. The interval for Directional Accuracy does not overlap that of any competing criterion.

**Criterion**	**Full-sample weight**	**Bootstrap median**	**95% CI**
RMSE	0.0222	0.0218	[0.0138, 0.0327]
MAE	0.0200	0.0197	[0.0124, 0.0300]
SMAPE	0.1486	0.1503	[0.0488, 0.2099]
DA	**0.8093**	**0.8077**	**[0.7379, 0.9130]**

S4.2 Results

Across the seven LOSO iterations, w_{DA} ranges from 0.7820 to 0.8375 (mean 0.8077) and retains the highest weight in *every* iteration; no perturbation reverses the criterion ranking (Table S4).

Under bootstrap resampling the 95% percentile interval for w_{DA} is [0.7379, 0.9130], which does not overlap the interval of SMAPE ([0.0488, 0.2099]) nor those of RMSE and MAE, and DA receives the highest weight in 100% of the 2,000 replicates (Table S5, Figure S2).

DA dominance is therefore not an artefact of the small matrix. Its structural cause is visible in the correlation matrix of the four criteria. RMSE and MAE are near-collinear ($r = 0.968$): both measure error magnitude in IQA units and differ only in how they penalise large residuals, so CRITIC's conflict term $\sum_{k \neq j}(1 - r_{jk})$ splits one dimension of information between them and assigns each a small share. SMAPE, although scale-relative, remains strongly associated with both ($r = 0.86$ and $r = 0.81$). DA is the only criterion in *conflict* with the error-magnitude block ($r = -0.41$ with RMSE, $r = -0.35$ with MAE, $r = -0.63$ with SMAPE) because it measures the sign of change rather than its size. Any objective weighting built on a conflict measure will concentrate weight on the criterion carrying non-redundant information; the *ordering* of the four weights follows from this correlation structure and is therefore a property of the criterion set, while the *magnitude* of w_{DA} depends on the sample and is bounded by the interval reported above.

S4.3 Effect on the reported results

The Block-3 and Block-4 layers were recomputed end-to-end from the raw agency archives for this revision, using the validated sub-index curves and the fixed regulatory weight schedule. The resulting model selection is Random Forest at three stations, XGBoost at two and LightGBM at two. The criterion ordering is unchanged from the original submission — Directional Accuracy first, then SMAPE, then the two error-magnitude criteria — and is now bounded by the resampling intervals reported above. What changes are the magnitudes, which the original submission understated, and the station count, which falls from nine to seven for the data-availability reasons set out in S2. The forecasting performance figures in Section 4.5 have been updated accordingly, and the adaptive-conformal coverage results are reported there on the same seven stations.

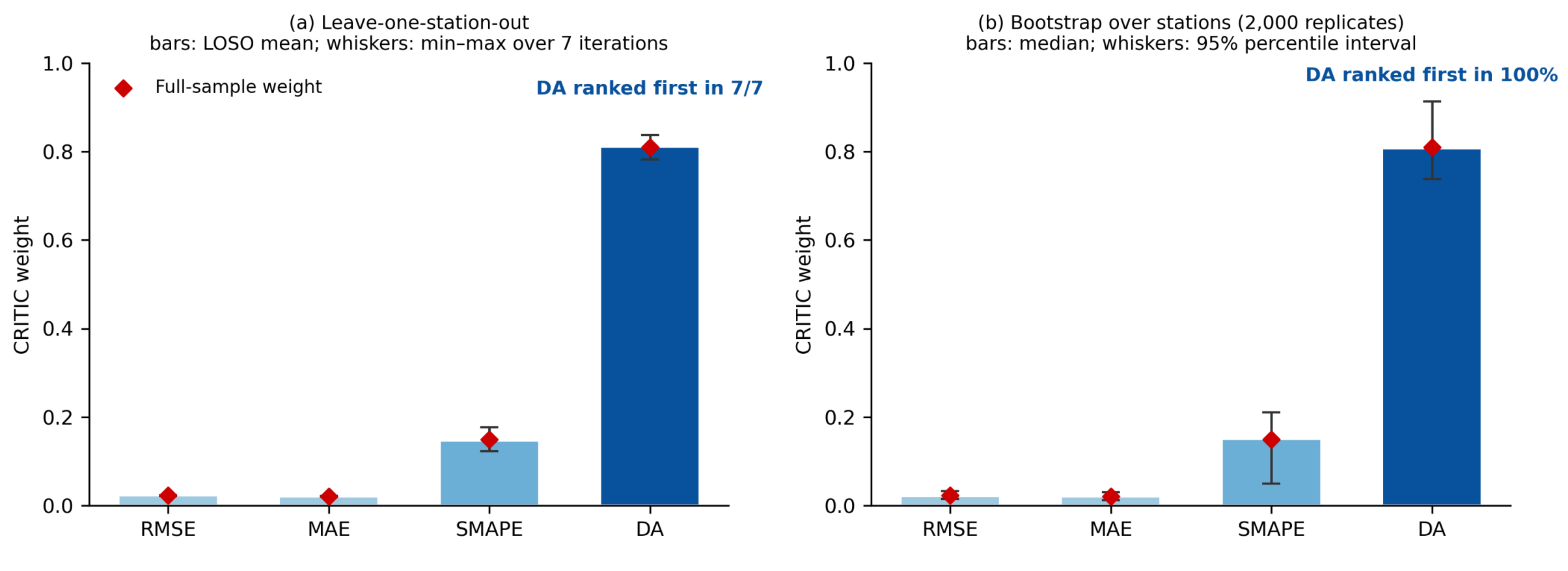

Figure S2: Stability of the Block-4 CRITIC criterion weights, computed on the 21-row station–model matrix. (a) Leave-one-station-out weights; bars show the LOSO mean, whiskers the minimum and maximum across the seven iterations, red diamonds the full-sample weight. (b) Bootstrap distribution over 2,000 station resamples; bars show the median, whiskers the 95% percentile interval, red diamonds the full-sample weight. Directional Accuracy retains the highest weight in every LOSO iteration and in every bootstrap replicate, and its interval does not overlap that of any competing criterion.

5 S5. Pooled versus basin-specific OWQI weight vectors

S5.1 Rationale

Reviewer 1 asked whether the OWQI weight vector reported in the main text is pooled across basins or basin-specific, and requested summary statistics if basin-specific vectors exist. The vector reported in the main text is derived from the pooled cross-basin q-matrix. For this revision we additionally derived independent weight vectors for each basin, applying the identical CRITIC–Shannon–MEREC game-theory procedure within each basin.

S5.2 Data scope of this analysis

This analysis is conducted on the full CETESB *Todos os Corpos Hídricos* archive rather than on the B1–B3 partition used in the main text, and the basins listed in Figure S3 therefore differ from those in Table 1 of the manuscript. Two considerations motivate this choice.

First, the question at issue is whether objective weighting is sensitive to basin context. Answering it requires as many hydrologically distinct basins as the data permit: the CETESB archive yields ten basins with sufficient nine-parameter coverage for independent weight derivation, against three in the main-text partition. A ten-basin comparison is a substantially stronger test of context-sensitivity than a three-basin one, and it spans a wider pressure gradient — from the Cantareira headwater tributaries to the heavily loaded Alto Tietê — than the three study basins alone.

Second, the two INEA-RJ basins (B2 Rio Guandu, B3 Baía de Guanabara) cannot support an equivalent derivation. B2 comprises only seven stations, and the nine-parameter simultaneous coverage required to construct the q-matrix is available for too few station-months in either basin to yield a stable weight vector. Reporting weight vectors derived from such sparse matrices would invite precisely the small-sample objection that Reviewer 1 correctly raised in respect of the Block-4 criterion weights (Supplementary S4).

The pooled vector reported in Table 2 of the main text is unaffected by this scope difference: it is derived from the B1–B3 partition as described in Section 3.2. The basin-specific vectors below are a robustness diagnostic addressing the reviewer's question about heterogeneity, not a restatement of the operational OWQI weights.

S5.3 Results and interpretation

The objective weighting consistently concentrates weight on the oxygen–sewage parameter group: dissolved oxygen and thermotolerant coliforms together receive between 0.278 and 0.412 of total weight in every basin, against 0.320 in the fixed IQA-CETESB schedule, and one of these two parameters ranks first in eight of the ten basins.

The identity of the single highest-weighted parameter is, however, basin-dependent. Thermotolerant coliforms rank first in five basins, dissolved oxygen in three (all three Tietê sub-basins), and turbidity in two (Capivari and Jundiaí). Dissolved oxygen weight varies almost twofold across basins (0.112–0.222). We therefore do *not* claim that a single parameter dominates universally. What transfers across basins is the structural finding that objective weighting redistributes weight away from the low-variance parameters fixed high in the regulatory schedule — pH receives 0.073–0.095 against a regulatory 0.120 in every basin without exception, and total solids 0.047–0.099 against 0.080 in eight of ten — and towards the parameters that actually discriminate between monitoring conditions.

The basin-level pattern is physically interpretable. Dissolved oxygen ranks first precisely in the Tietê sub-basins, where sustained organic loading from the São Paulo metropolitan region drives oxygen sag and makes DO the discriminating variable; turbidity ranks first in Capivari and Jundiaí, where episodic sediment loading dominates variability. This basin-dependence supports

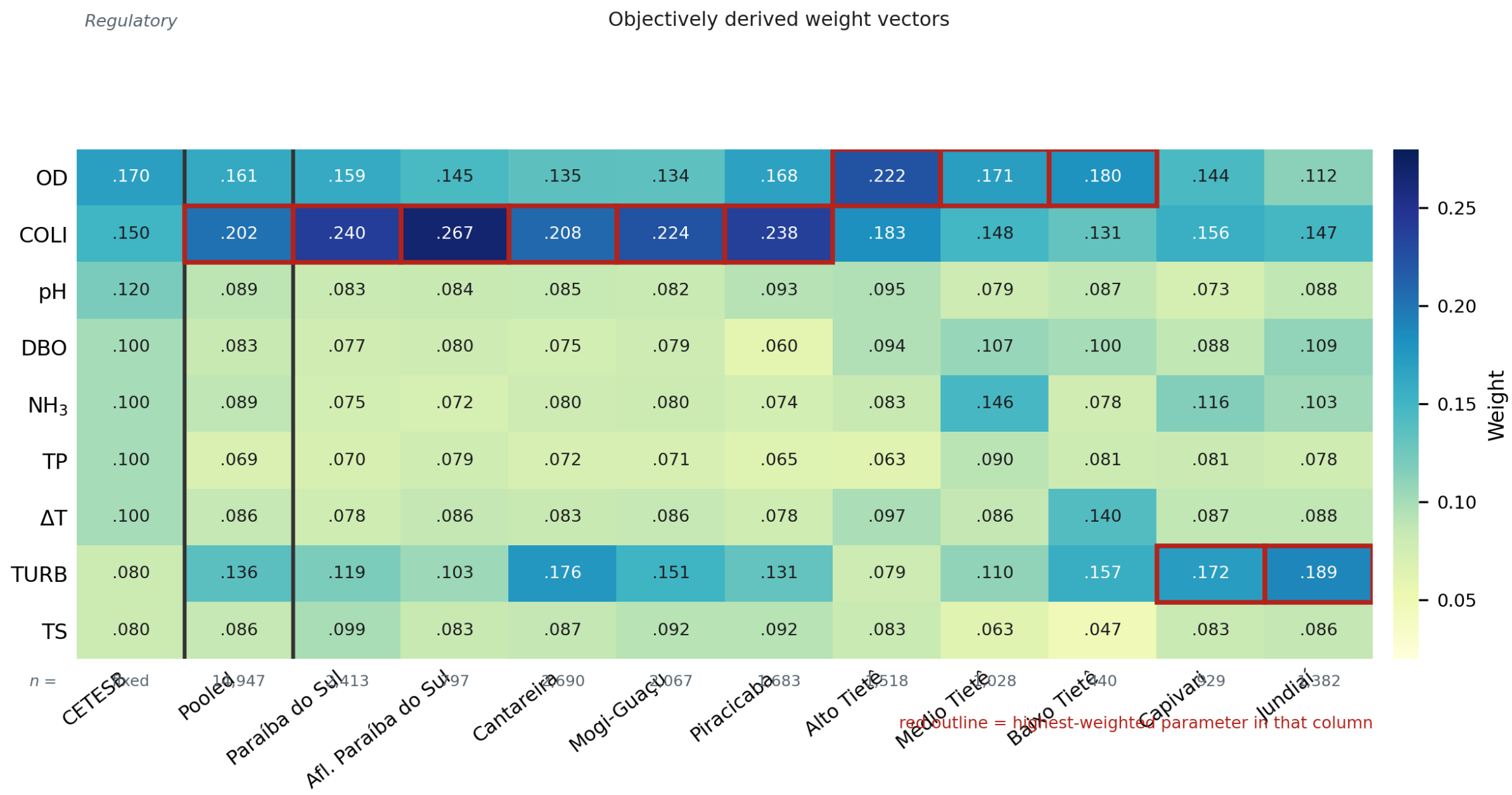

Figure S3: Game-theory (OWQI) parameter weight vectors derived independently per basin, compared with the pooled vector and the fixed CETESB regulatory schedule. Rows are the nine IQA parameters; columns are the regulatory reference, the pooled cross-basin vector, and the ten basins with sufficient nine-parameter coverage for independent derivation. Cell values are weights; the red outline marks the highest-weighted parameter within each objectively derived vector. n denotes complete nine-parameter station-months. Thermotolerant coliforms lead in five basins, dissolved oxygen in the three Tietê sub-basins, and turbidity in Capivari and Jundiaí, while pH and total solids remain below their regulatory weights in every basin.

the paper's central argument: a fixed national weight schedule cannot represent the discriminating structure of hydrologically distinct basins, which is the case for data-driven weighting rather than against it.

6 S6. Reproducibility

All analyses in this Supplementary Material were computed directly from the CETESB *Todos os Corpos Hídricos* archive (646,356 raw records, 141 stations, 369 raw parameter nomenclatures). Harmonisation to the nine canonical IQA identifiers, IQA computation via the CETESB multiplicative formulation with Benson–Krause oxygen saturation, the objective weighting routines (CRITIC, Shannon entropy, MEREC, game-theory aggregation), the forecasting benchmark and the resampling diagnostics are implemented in the analysis scripts released with the manuscript.
